# Effect of single-dose, live, attenuated dengue vaccine in children with or without previous dengue on risk of subsequent, virologically confirmed dengue in Cebu, the Philippines: a longitudinal, prospective, population-based cohort study

**DOI:** 10.1016/S1473-3099(24)00099-9

**Published:** 2024-07

**Authors:** Michelle Ylade, Maria Vinna Crisostomo, Jedas Veronica Daag, Kristal An Agrupis, Anna Maureen Cuachin, Ava Kristy Sy, Deok Ryun Kim, Hyeon Seon Ahn, Ana Coello Escoto, Leah C Katzelnick, Cameron Adams, Laura White, Aravinda M de Silva, Jacqueline Deen, Anna Lena Lopez

**Affiliations:** a Institute of Child Health and Human Development, National Institutes of Health, University of the Philippines Manila, Manila, Philippines; b Department of Virology, Research Institute for Tropical Medicine, Muntinlupa, Philippines; c International Vaccine Institute, Seoul, South Korea; d Viral Epidemiology and Immunity Unit, National Institute of Allergy and Infectious Diseases, National Institutes of Health, Bethesda, MD, USA; e Department of Microbiology and Immunology, University of North Carolina School of Medicine, Chapel Hill, NC, USA; f Institute of Child Health and Human Development, National Institutes of Health, University of the Philippines Manila, Manila, Philippines

## Abstract

**Background:**

A three-dose dengue vaccine (CYD-TDV) was licensed for use in children aged 9 years and older starting in 2015 in several dengue-endemic countries. In 2016, the Philippine Department of Health implemented a dengue vaccination programme, which was discontinued because of safety concerns. We assessed the relative risk of developing virologically confirmed dengue among children who did or did not receive a single dose of CYD-TDV by previous dengue virus (DENV) infections at baseline classified as none, one, and two or more infections.

**Methods:**

In this longitudinal, prospective, population-based cohort study, we enrolled healthy children (aged 9–14 years) residing in Bogo or Balamban, Cebu, Philippines, between May 2, and June 2, 2017, before a mass dengue vaccination campaign, via the Rural Health Unit in Bogo and three Rural Health Units in Balamban. We collected demographic information and sera for baseline DENV serostatus and conducted active surveillance for acute febrile illness. Children who developed acute febrile illness were identified, clinical data were collected, and blood was drawn for confirmation of dengue by RT-PCR. The primary outcome was the relative risk of developing virologically confirmed dengue among children who received or did not receive a single dose of CYD-TDV by DENV serostatus at baseline.

**Findings:**

A single dose of CYD-TDV did not confer protection against virologically confirmed dengue in children who had none or one previous DENV infection at baseline. One dose conferred significant protection against hospital admission for virologically confirmed dengue among participants who had two or more previous DENV infections at baseline during the first 3 years (70%, 95% CI 20–88; p=0·017) and the entire follow-up period (67%, 19–87; p=0·016).

**Interpretation:**

The risk of developing virologically confirmed dengue after a single dose of CYD-TDV varied by baseline DENV serostatus. Since the study assessed the effect of only a single dose, the findings cannot inform decisions on vaccination by public health officers. However, the findings have implications for children who receive an incomplete vaccination regimen and these results should prompt more detailed analyses in future trials on dengue vaccines.

**Funding:**

The Philippine Department of Health, Hanako Foundation, WHO, Swedish International Development Cooperation Agency, International Vaccine Institute, University of North Carolina, and US National Institute of Allergy and Infectious Diseases.

## Introduction

There are four serotypes of dengue virus (DENV) and repeat infections can occur.^1^ Dengue can be asymptomatic, manifest with or without warning signs, or manifest as a severe illness.^2^ Secondary DENV infection with a heterotypic serotype is associated with an increased risk for serious illness compared with primary infection^3^ but confers broadly protective immunity such that post-secondary infections are generally mild.^4^, ^5^

In 2015, a three-dose dengue vaccine (CYD-TDV, Dengvaxia, Sanofi Pasteur, Lyon, France)^6^ was licensed in several dengue-endemic countries in people aged 9 years and older. In clinical trials, CYD-TDV performed worse in younger children than older children because, as was later shown, DENV seropositivity increased with age.^7^, ^8^ In 2016, the Philippine Department of Health implemented a dengue vaccination programme among children in Central Luzon, Calabarzon, and Metro Manila, which was extended to Cebu Province. Further analysis of the CYD-TDV phase 3 trial showed that the vaccine was contraindicated in individuals seronegative for DENV.^9^ The Philippine dengue vaccination programme was, therefore, discontinued and children in Cebu were offered only one dose.

**Research in context**

**Evidence before this study**

When this study was designed, CYD-TDV (Dengvaxia, Sanofi Pasteur, Lyon, France) was being rolled out in mass campaigns in children aged 9–14 years in the Philippines. After Sanofi Pasteur announced the relabelling of the vaccine, the dengue vaccination programme was abruptly discontinued, such that only a single dose was offered in the last vaccination site, Cebu Province. We searched PubMed for publications from Jan 1, 2010, to Nov 19, 2023, with the terms “CYD-TDV”, “Dengvaxia”, “single-dose”, “immune*”, and “antibody”, without any language restrictions or inclusion criteria. The search retrieved three articles reporting protection conferred by a single dose of CYD-TDV. A post-hoc analysis of the CYD-TDV phase 3 trials assessed the efficacy of CYD-TDV during the 6-month period between the first and second dose. The estimated vaccine efficacy in participants aged 9 years or older who tested seropositive at 6 months post-dose one was 81%, whereas there was null to modest protection in participants who were seronegative. In a case–control study in Cebu, Philippines, one dose of CYD-TDV was associated with 48% protection against virologically confirmed dengue with warning signs and severe virologically confirmed dengue about 2 years after mass vaccination in children aged 9–14 years. A case–control study in Parana, Brazil, found a single dose of CYD-TDV provided 26% protection against virologically confirmed dengue in individuals aged 19–27 years, and 86% protection against only dengue virus (DENV)4 within 3 years following mass vaccination. These studies could not evaluate the vaccine effect in children with previous DENV infection classified as one versus two or more infections.

**Added value of this study**

It was previously thought that people with one past DENV infection at baseline (monotypic profile) would benefit most from CYD-TDV, protecting them from a severe second DENV infection. By comparison, individuals with two or more past DENV infections (multitypic profile) would already have protective immunity. However, in the immunogenicity subset of the three-dose CYD-TDV phase 3 trial, a lower vaccine efficacy point estimate against virologically confirmed dengue requiring hospital admission was already noted in individuals with a monotypic profile (75·3%) than in individuals with a multitypic profile (81·2%). We found that no protection was conferred by a single dose of Dengvaxia to those with a naive or monotypic DENV immune profile at baseline but was protective against virologically confirmed dengue requiring hospital admission among those with a multitypic profile at baseline.

**Implications of all the available evidence**

Since only a single dose was given, this study cannot be used to inform decisions on vaccination by public health officials but it has implications for children who receive an incomplete vaccination regimen. Our results suggest that participant baseline DENV serostatus in future dengue vaccine trials should ideally be analysed by the number of previous DENV infections (not just as DENV seropositive or seronegative) to provide detailed knowledge of the outcome of vaccination.

We aimed to assess the relative risk of developing virologically confirmed dengue among children in Cebu who received or did not receive a single dose of CYD-TDV, by the number of previous DENV infections (none, one, or two or more) at baseline.

## Methods

### Study design and participants

In this longitudinal, prospective, population-based cohort study, we invited healthy children aged 9–14 years (at the time of the campaign) residing in Bogo city and Balamban municipality to participate in the study between May 2 and June 2, 2017, before the mass dengue vaccination campaign in Cebu.^10^ Children were recruited and followed up through the Rural Health Unit in Bogo and three Rural Health Units in Balamban. A parent or legal guardian of the participants provided written informed consent. Verbal assent was obtained from the participants and documented.^10^ This study followed the STROBE guidelines^11^ and the protocol (appendix pp 2–35) was approved by the University of the Philippines Manila research ethics board.

### Procedures

Participants completed a questionnaire on demographic information and provided a 5 mL venous blood sample to measure baseline DENV serostatus as previously described.^10^ Rural Health Units prepared a master list with the name, date of birth, sex, and address of healthy children invited to participate in the campaign. The master list included 285 242 children, of whom 149 023 (52·2%) received a dose of CYD-TDV from June 15 to Aug 16, 2017. A vaccination card was given to each vaccinated participant and the master list was maintained as the vaccination registry. The dengue vaccination programme was discontinued in late 2017 and no further doses were offered in Cebu.

2996 children were enrolled before mass vaccination. We ascertained subsequent receipt of CYD-TDV in face-to-face interviews and by checking vaccination cards. For participants who claimed to have been vaccinated but did not have a card, vaccination status was confirmed by linkage to the registry based on name, date of birth, sex, and address, without knowledge of subsequent detection of virologically confirmed dengue in participants. Socioeconomic, behavioural, and environmental variables were ascertained through questionnaires administered to the participants and families.

During enrolment, we provided each participant and the parent or guardian with a digital thermometer and instructions on whom to contact in case of acute febrile illness. All participants in this fixed cohort, regardless of baseline serostatus and receipt of CYD-TDV, were actively monitored via monthly contact with the parents or guardians, who were reminded to call study staff in case of febrile illness (temperature ≥38°C on ≥2 consecutive days) and to take the child to a health-care centre for management according to national guidelines. During the participant's illness, we completed a standard questionnaire that included clinical signs and symptoms and obtained a 5 mL blood sample. Blood samples were collected in anticoagulant-free vacutainer tubes, processed, and aliquoted. Sera were frozen at –20°C and shipped in dry ice to the Research Institute of Tropical Medicine. Total nucleic acid was extracted from serum samples using the QIAmp Viral RNA kit (QIAGEN, Valencia, CA, USA) according to the manufacturer's protocol. DENV was typed using multiplex single-step real-time RT-PCR as described previously.^12^

A participant with virologically confirmed dengue was defined as having acute febrile illness with a positive result by RT-PCR. We interviewed participants or the parent or guardian in hospital or at home in person or via telephone to establish the outcome of the illness. We classified cases of virologically confirmed dengue according to WHO criteria.^13^ Surveillance was continued during varying levels of COVID-19 lockdown across the Philippines, starting in March, 2020 and ending in September, 2021.^14^

All participants were invited to provide follow-up blood samples between October, 2018, and September, 2019 (17–28 months after baseline samples were obtained). We compared neutralising antibody concentrations in paired baseline and follow-up samples from vaccinated and unvaccinated participants with DENV naive, monotypic, and multitypic profiles at baseline. The inclusion criteria were based on the availability of paired baseline or follow-up samples. Since the number of participants classified as having a multitypic profile at baseline was too large to test, representative subgroups of 10% were randomly selected using the sample function of R (version 4.2.1).

Neutralising antibody concentrations were measured using a representative mature, low-passage clinical strain circulating in southeast Asia for each serotype. Mature dengue virions (GenBank numbers, appendix p 36) were produced by infecting Vero cells overexpressing human furin.^15^ Serial 3-fold to 4-fold dilutions of heat-inactivated serum were mixed with a constant amount (60–90 foci per well) of each of the four DENV virus serotypes. Mixtures were inoculated into wells of a 96-well plate of confluent Vero cells. After adsorption, cells were overlayed with carboxymethylcellulose or methylcellulose and incubated for 44–52 h, then fixed and immunostained with DENV E and prM crossreactive monoclonal antibodies 2H2 (ATCC HB-112; 1·63 mg/mL; Lofstrand Labs, Gaithersburg, MD, USA;) or 4G2 (ATCC HB-112; 0·8 mg/mL; Lofstrand Labs), or both. Foci were counted in each well and compared with foci count in control virus-only wells.

### Statistical analysis

The primary outcome was the relative risk of developing virologically confirmed dengue among children who received or did not receive a single dose of CYD-TDV by DENV serostatus at baseline. We compared participant characteristics by DENV serostatus at baseline and receipt of CYD-TDV. p values in vaccinated and unvaccinated children were derived using a two-sample *t* test and Wilcoxon rank-sum test for mean and median comparisons, respectively. p values in participants with DENV naive, monotypic, and multitypic profiles were derived using a one-way ANOVA test and Kruskal-Wallis test for mean and median comparisons, respectively. We calculated the overall cumulative incidence per 100 person-years (incidence rate) of the first episodes of acute febrile illness and virologically confirmed dengue in the cohort by year of observation. The start of observation was the date of receipt of CYD-TDV for vaccinated children and the median date of CYD-TDV roll-out in Cebu for unvaccinated children. The end of observation was Oct 31, 2022, for children who completed the entire surveillance period or the last follow-up date for children who migrated, died, or were lost to follow-up. We estimated the crude relative risk of developing virologically confirmed dengue (all and by severity classification) and hospital admission due to virologically confirmed dengue by vaccination status and baseline dengue serostatus for the first 3 years and the entire follow-up period as hazard ratio (HR). We adjusted for variables that differed significantly between participants who received the vaccine and those who did not using Cox proportional hazards models. Variables were selected using forward step-wise methods for these models after verifying that the proportionality assumption was fulfilled using weighted Schoenfeld residuals, p value for entry was less than 0·1, and p value for retention was less than 0·05.^16^ The HR was estimated by exponentiating the coefficient for the variable of vaccination status group and the SE of this coefficient was used to estimate the p value and 95% CIs for HR. When the HR was less than 1, vaccine protection was calculated as (1–HR) × 100%. The cumulative incidence rate of virologically confirmed dengue according to baseline DENV serostatus is shown on Kaplan–Meier curves. Results were presented for the first 3 years and the entire follow-up period because of the decrease in acute febrile episodes and DENV infections during the COVID-19 lockdown, also described in other settings.^17^ Statistical analyses were performed using SAS (version 9.4).

The secondary outcome was the immune response to DENV1–4 in vaccinated and unvaccinated participants, by DENV serostatus at baseline. Half maximal effective concentration values were calculated by graphing percentage neutralisation versus serum dilution and fitting a sigmoidal dose–response curve with a variable slope (R^2^ >0·75 and a hill slope >0·5 absolute value) using GraphPad Prism (version 9) or R (version 4.2.1). Neutralising antibody titres less than a 1/20 serum dilution were below the assay limit of detection and were considered negative and tabulated as 10. Log transformed titres were used to calculate geometric mean titre for each serotype with 95% CIs. Seropositivity was defined as the proportion of participants with neutralisation above the assay limit of detection. A paired *t* test was performed to compare baseline to follow-up titres against each serotype.

The sample size of this longitudinal cohort study was based on its objective to determine the relative risk of developing virologically confirmed dengue among children who received or did not receive CYD-TDV, by dengue serostatus at baseline (appendix pp 2–30), and was calculated using the following assumptions: 80% power, α of 5%, dengue seroprevalence of 80%, and a relative risk of 3·5 of developing DENV disease in children who were seronegative versus children who were seropositive.^10^

This study is registered with ClinicalTrials.gov, NCT03465254.

### Role of the funding source

The funders of the study had no role in study design, data collection, data analysis, data interpretation, or writing of the report.

## Results

**Figure 1:**
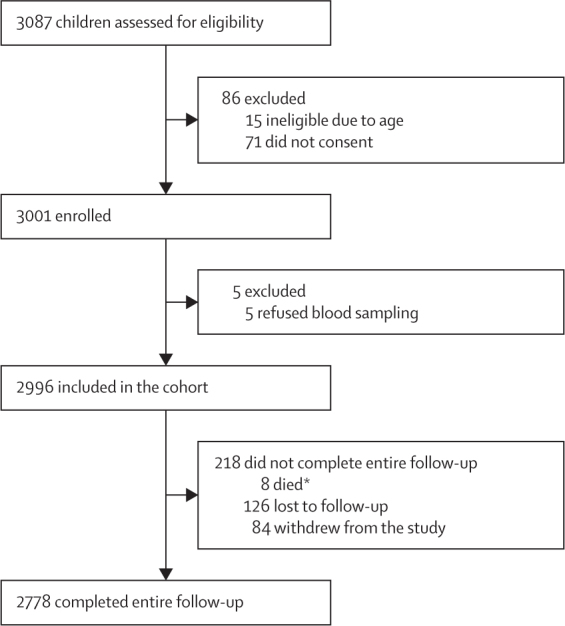
Trial profile *All deaths had baseline dengue virus multitypic profiles (possible sepsis [unvaccinated], meningitis [one-dose CYD-TDV], motor accident [unvaccinated], myopathy [unvaccinated], two suicides [unvaccinated], malignancy [one-dose CYD-TDV], and pneumonia [one-dose CYD-TDV]).

Between May 2 and June 2, 2017, of 3087 children assessed for eligibility, 3001 (97·2%) were enrolled into the study and baseline sera were collected from 2996 (97·1%). 2778 children completed the entire period of surveillance for acute febrile illness (figure 1). Mean age was 10·9 years (SD 1·4) and of 2996 children, 1545 (51·6%) were female and 1451 (48·5%) were male (table 1). Older age and residence in Bogo city, which is more urbanised than the Balamban municipality, was associated with having a DENV multitypic profile at baseline (p<0·0001; appendix p 37). Of 2996 children, 320 (10·7%) had a DENV naive profile, 292 (9·7%) had a monotypic DENV profile (indicative of one past infection), and 2384 (79·6%) had a multitypic DENV profile (evidence of at least two different dengue serotypes causing previous infection). 1790 (59·7%) of 2996 children received a single dose of CYD-TDV. Participants who lived in Bogo city (p=0·0035) and those whose households owned a radio (p=0·016) or a bicycle (p=0·054) were significantly more likely to have received CYD-TDV (table 1).

**Table 1:** Baseline characteristics

	**Vaccinated (one dose; n=1790)**	**Unvaccinated (n=1206)**	**Total (n=2996)**	p value*
**Age (years) at enrolment**				
Mean (SD)	10·9 (1·4)	10·9 (1·4)	10·9 (1·4)	0·57
Median (IQR)	11 (8–15)	11 (8–15)	11 (8–15)	0·57
**Sex**				
Female	916 (51·2%)	629 (52·2%)	1545 (51·6%)	0·60
Male	874 (48·9%)	577 (47·9%)	1451 (48·5%)	..
**Residence**				
Bogo city	970 (54·2%)	588 (48·8%)	1558 (52·0%)	0·0035
Balamban municipality	820 (45·8%)	618 (51·3%)	1438 (48·0%)	..
**Baseline dengue serostatus**				
Dengue naive	182 (10·2%)	138 (11·5%)	320 (10·7%)	0·27
Dengue monotypic profile	170 (9·5%)	122 (10·1%)	292 (9·8%)	0·58
Dengue multitypic profile	1438 (80·3%)	946 (78·4%)	2384 (79·6%)	0·21
**Uses house screen**†				
Yes	173 (10·5%)	123 (12.0%)	296 (11·1%)	0·25
No	1470 (89·5%)	903 (88·0%)	2373 (88·9%)	..
**Housing material**†				
Wood	799 (48·6%)	503 (49·0%)	1302 (48·8%)	0·87
Cement or concrete	844 (51·4%)	523 (51·0%)	1367 (51·2%)	..
**Keeps containers and unused tyres**†				
Yes	199 (12·1%)	125 (12·2%)	324 (12·2%)	0·95
No	1444 (87·9%)	901 (87·8%)	2345 (87·9%)	..
**Number of individuals in the household**†				
Mean (SD)	5·7 (1·9)	5·8 (2·1)	5·7 (2.0)	0·44
Median (IQR)	5 (2–15)	5 (1–24)	5 (1–24)	0·93
**Number of children within the household**†				
Mean (SD)	2·6 (1·4)	2·7 (1·6)	2·7 (1·5)	0·091
Median (IQR)	2 (0– 8)	2 (0–14)	2 (0–14)	0·60
**Household head with >6 years of schooling**†				
Yes	1571 (95·6%)	971 (94·6%)	2542 (95·2%)	0·26
No	72 (4·4%)	55 (5·4%)	127 (4·8%)	..
**Migrated to current residence in past 2 years**†				
Yes	42 (2·6%)	28 (2·7%)	70 (2·6%)	0·80
No	1601 (97·4%)	998 (97·3%)	2599 (97·4%)	..
**Ownership of items**†				
Radio	748 (45·5%)	418 (40·7%)	1166 (43·7%)	0·016
Television	1231 (74·9%)	756 (73·7%)	1987 (74·5%)	0·49
Refrigerator	814 (49·5%)	546 (53·2%)	1360 (51.0%)	0·067
Bicycle	500 (30·4%)	276 (26·9%)	776 (29·1%)	0·054
Motorcycle	946 (57·6%)	588 (57·3%)	1534 (57·5%)	0·90
Mobile phone	1621 (98·7%)	1005 (98.0%)	2626 (98·4%)	0·16
Desktop or handheld computer	266 (16·2%)	166 (16·2%)	432 (16·2%)	1·00
Car	83 (5·1%)	63 (6·1%)	146 (5·5%)	0·26
**Ownership of all luxury items**†‡				
Yes	56 (3·4%)	45 (4·4%)	101 (3·8%)	0·21
No	1587 (96·6%)	981 (95·6%)	2568 (96·2%)	..
**Ownership of at least one luxury item**†‡				
Yes	1633 (99·4%)	1012 (98·6%)	2645 (99·1%)	0·057
No	10 (0·6%)	14 (1·4%)	24 (0·9%)	..
**Estimated monthly household expenditure,** ₱†				
Mean (SD)	11 484·4 (5663·3)	11 772·0 (7163·7)	11 595·0 (6282·8)	0·28
Median (IQR)	10 000 (1000–60 000)	10 000 (2000–100 000)	10 000 (1000–100 000)	0·98
**Use of topical insect repellant**†				
Yes	130 (7·9%)	78 (7·6%)	208 (7·8%)	0·82
No	1512 (92·0%)	948 (92·4%)	2460 (92·2%)	0·77
Unknown	1 (0·1%)	0	1 (0·0%)	1·00
**Use of burned mosquito coil**†				
Yes	231 (14·1%)	126 (12·3%)	357 (13·4%)	0·20
No	1412 (85·9%)	900 (87·7%)	2312 (86·6%)	..
**Fogging in the past month**†				
Yes	2 (0·1%)	5 (0·5%)	7 (0·3%)	0·12
No	1639 (99·8%)	1019 (99·3%)	2658 (99·6%)	0·12
Unknown or NA	2 (0·1%)	2 (0·2%)	4 (0·2%)	0·64

NA=not applicable. ₱=Philippine pesos. US$1·00=₱55·92.

* p value has been derived using a two-sample *t* test and Wilcoxon rank-sum test for mean and median comparison, respectively, for continuous variables, and χ^2^ and Fisher's exact test for binary variables.

† Data are missing for 327 participants.

‡ Luxury items include a car, computer, mobile phone, refrigerator, and television.

Follow-up serum samples were obtained from 2375 (79·3%) of 2996 participants. Of 2375 participants, 241 (10·1%) had a DENV naive profile (148 vaccinated and 93 unvaccinated), 228 (9·6%) had a DENV monotypic profile (145 vaccinated and 83 unvaccinated), and 1906 (80·3%) had a DENV multitypic profile (1250 vaccinated and 656 unvaccinated) at baseline. Surveillance was carried out from Nov 6, 2017, to Oct 31, 2022. Of 2996 participants, 218 (7·3%) did not complete the entire follow-up (eight died, 126 were lost to follow-up, and 84 withdrew; figure 1). During the surveillance period, 674 participants had a first episode of acute febrile illness, of whom 153 (22·7%) had virologically confirmed dengue (73 without warning signs, 80 with warning signs; none with severe dengue, 36 requiring hospital admission). DENV serotypes are shown in the appendix (p 39). For the whole surveillance period, the cumulative incidence rate was 4·84 cases per 100 person-years (95% CI 4·50–5·21) for acute febrile episodes, 1·02 cases per 100 person-years (0·87–1·19) for virologically confirmed dengue, 0·48 cases per 100 person-years (0·39–0·61) for virologically confirmed dengue without warning signs, 0·53 cases per 100 person-years (0·43–0·66) for virologically confirmed dengue with warning signs, and 0·24 cases per 100 person-years (0·17–0·33) for virologically confirmed dengue requiring hospital admission (appendix p 40). The incidence rate of virologically confirmed dengue was 1·81 (1·25–2·61) among participants with a DENV naive profile, 1·74 (1·18–2·56) in those with a DENV monotypic profile, and 0·83 (0·68–1·01) in those with a DENV multitypic profile at baseline (appendix p 41).

We calculated the crude and adjusted relative risk estimates of developing virologically confirmed dengue (any, without warning signs, with warning signs, and requiring hospital admission) by vaccination status and baseline DENV serostatus during the first 3 years and the entire follow-up period (Table 2, Table 3). Among participants who were DENV naive or had a monotypic profile at baseline, the crude and adjusted HR (vaccinated *vs* unvaccinated) was greater than 1 for virologically confirmed dengue (any, without, and with warning signs) during the first 3 years and the entire follow-up period, although this difference was not significant. Among participants with a DENV naive profile, hospital admission for virologically confirmed dengue was only required among vaccinated individuals.

**Table 2:** Crude and adjusted relative risk estimates of developing VCD by vaccination status and baseline dengue serostatus for 3-year follow-up

		**Vaccinated (one dose)**		**Unvaccinated**		**Crude**		**Adjusted**	
		n; cases	Incidence rate (95% CI)	n; cases	Incidence rate (95% CI)	HR (95%CI)	p value	HR (95%CI)	p value
Any VCD		1790; 84	1·60 (1·29–1·98)	1206; 51	1·44 (1·10–1·90)	1·11 (0·78– 1·57)	0·56	1·01 (0·71–1·43)	0·95*
	Dengue naive	182; 17	3·24 (2·03–5·17)	138; 9	2·28 (1·19–4·34)	1·42 (0·63–3·19)	0·39	1·42 (0·63–3·19)	0·39
	Dengue monotypic profile	170; 14	2·83 (1·69–4·75)	122; 9	2·54 (1·33–4·85)	1·11 (0·48–2·57)	0·80	1·28 (0·55–2·98)	0·56†
	Dengue multitypic profile	1438; 53	1·25 (0·96–1·64)	946; 33	1·19 (0·84–1·66)	1·06 (0·68–1·63)	0·81	1·06 (0·68–1·63)	0·81
VCD without warning signs		1790; 43	0·82 (0·61–1·10)	1206; 19	0·54 (0·34–0·84)	1·52 (0·89–2·61)	0·13	1·36 (0·79–2·33)	0·27*
	Dengue naive	182; 9	1·71 (0·90–3·27)	138; 4	1·01 (0·38–2·68)	1·69 (0·52–5·48)	0·38	1·55 (0·48–5·02)	0·47*
	Dengue monotypic profile	170; 5	1·01 (0·42–2·42)	122; 3	0·85 (0·27–2·61)	1·19 (0·28–4·99)	0·81	1·19 (0·28–4·99)	0·81
	Dengue multitypic profile	1438; 29	0·68 (0·48–0·98)	946; 12	0·43 (0·25–0·76)	1·59 (0·81–3·11)	0·18	1·40 (0·71–2·74)	0·33*
VCD with warning signs		1790; 41	0·78 (0·58–1·06)	1206; 32	0·91 (0·64–1·28)	0·86 (0·54–1·37)	0·53	0·82 (0·51–1·30)	0·39‡
	Dengue naive	182; 8	1·52 (0·77–3·03)	138; 5	1·26 (0·53–3·02)	1·21 (0·39–3·69)	0·74	1·21 (0·39–3·69)	0·74
	Dengue monotypic profile	170; 9	1·82 (0·95–3·48)	122; 6	1·69 (0·77–3·75)	1·07 (0·38–3·02)	0·89	1·07 (0·38–3·02)	0·89
	Dengue multitypic profile	1438; 24	0·57 (0·38–0·84)	946; 21	0·75 (0·49–1·16)	0·75 (0·42–1·35)	0·34	0·75 (0·42–1·35)	0·34
Hospitalised due to VCD		1790; 16	0·30 (0·19–0·50)	1206; 17	0·48 (0·30–0·77)	0·63 (0·32–1·25)	0·19	0·61 (0·31–1·20)	0·15‡
	Dengue naive	182; 4	0·76 (0·29–2·02)	138; 0	NA	NA	NA	NA	NA
	Dengue monotypic profile	170; 6	1·21 (0·55–2·69)	122; 4	1·13 (0·43–2·99)	1·08 (0·30–3·83)	0·90	1·17 (0·33–4·16)	0·80§
	Dengue multitypic profile	1438; 6	0·14 (0·06–0·32)	946; 13	0·47 (0·27–0·80)	0·30 (0·12–0·80)	0·016	0·30 (0·12–0·80)	0·016

Years were calculated on the basis of the vaccination date for vaccinated individuals and median vaccination date for those not vaccinated. Incidence rate is per 100 person-years. HR=hazard ratio. NA=not applicable. VCD=virologically confirmed dengue.

* Adjusted for owning bicycle.

† Adjusted for residence and owning refrigerator.

‡ Adjusted for owning refrigerator.

§ Adjusted for residence.

**Table 3:** Crude and adjusted relative risk estimates of developing VCD by vaccination status and baseline dengue serostatus for entire follow-up

		**Vaccinated (one dose)**		**Unvaccinated**		**Crude**		**Adjusted**	
		n; cases	Incidence rate (95% CI)	n; cases	Incidence rate (95% CI)	HR (95%CI)	p value	HR (95%CI)	p value
Any VCD		1790; 96	1·06 (0·87–1·29)	1206; 57	0·95 (0·73–1·23)	1·13 (0·81–1·56)	0·48	1·05 (0·76–1·46)	0·76*
	Dengue naive	182; 18	2·02 (1·28–3·19)	138; 10	1·52 (0·82–2·82)	1·35 (0·62–2·92)	0·45	1·22 (0·56–2·64)	0·62†
	Dengue monotypic profile	170; 16	1·90 (1·17–3·08)	122; 9	1·51 (0·79–2·89)	1·27 (0·56–2·87)	0·57	1·43 (0·63–3·25)	0·40‡
	Dengue multitypic profile	1438; 62	0·85 (0·66–1·08)	946; 38	0·80 (0·58–1·10)	1·07 (0·71–1·60)	0·75	1·07 (0·71–1·60)	0·75
VCD without warning signs		1790; 50	0·55 (0·42–0·73)	1206; 23	0·38 (0·25–0·58)	1·45 (0·89–2·38)	0·14	1·31 (0·80–2·14)	0·29†
	Dengue naive	182; 10	1·12 (0·61–2·08)	138; 5	0·76 (0·32–1·82)	1·49 (0·51–4·35)	0·47	1·39 (0·47–4·06)	0·55†
	Dengue monotypic profile	170; 6	0·71 (0·32–1·58)	122; 3	0·50 (0·16–1·56)	1·42 (0·36–5·69)	0·62	1·42 (0·36–5·69)	0·62
	Dengue multitypic profile	1438; 34	0·46 (0·33–0·65)	946; 15	0·32 (0·19–0·52)	1·48 (0·81–2·72)	0·21	1·32 (0·72–2·43)	0·37†
VCD with warning signs		1790; 46	0·51 (0·38–0·68)	1206; 34	0·57 (0·40–0·79)	0·91 (0·58–1·41)	0·66	0·91 (0·58–1·41)	0·66
	Dengue naive	182; 8	0·90 (0·45–1·79)	138; 5	0·76 (0·32–1·82)	1·21 (0·39–3·69)	0·74	1·21 (0·39–3·69)	0·74
	Dengue monotypic profile	170; 10	1·19 (0·64–2·20)	122; 6	1·01 (0·45–2·23)	1·19 (0·43–3·27)	0·74	1·19 (0·43–3·27)	0·74
	Dengue multitypic profile	1438; 28	0·38 (0·26–0·55)	946; 23	0·48 (0·32–0·73)	0·80 (0·46–1·38)	0·42	0·80 (0·46–1·38)	0·42
Hospitalised due to VCD		1790; 18	0·20 (0·13–0·32)	1206; 18	0·30 (0·19–0·47)	0·67 (0·35–1·29)	0·23	0·64 (0·34–1·24)	0·19§
	Dengue naive	182; 4	0·45 (0·17–1·19)	138; 0	NA	NA	NA	NA	NA
	Dengue monotypic profile	170; 7	0·83 (0·40–1·74)	122; 4	0·67 (0·25–1·78)	1·25 (0·37–4·28)	0·72	1·34 (0·39–4·58)	0·64§
	Dengue multitypic profile	1438; 7	0·10 (0·05–0·20)	946; 14	0·29 (0·17–0·50)	0·33 (0·13–0·81)	0·016	0·33 (0·13–0·81)	0·016

Years were calculated on the basis of vaccination date for vaccinated individuals and median vaccination date for those who were not vaccinated. Incidence rate is per 100 person-years. HR=hazard ratio. NA=not applicable. VCD=virologically confirmed dengue.

* Adjusted for owning refrigerator and bicycle.

† Adjusted for owning bicycle.

‡ Adjusted for residence and owning refrigerator.

§ Adjusted for residence.

By contrast, among participants with a DENV multitypic profile at baseline, the crude and adjusted HR (vaccinated *vs* unvaccinated) was less than 1 for virologically confirmed dengue with warning signs or requiring hospital admission during the first 3 years and the entire follow-up period. The adjusted HR for virologically confirmed dengue with warning signs was 0·75 (95% CI 0·42–1·35, p=0·34) over 3 years and 0·80 (0·46–1·38, p=0·42) during the entire follow-up period. The adjusted HR for virologically confirmed dengue requiring hospital admission was 0·30 (0·12–0·80, p=0·016) over 3 years and 0·33 (0·13–0·81, p=0·016) during the entire follow-up period. A single dose of CYD-TDV conferred significant protection against dengue requiring hospital admission in those with a DENV multitypic profile at baseline (Table 2, Table 3). The adjusted vaccine protection against dengue requiring hospital admission in the participants with a multitypic profile was 70% (95% CI 20–88; p=0·017) during the first 3 years and 67% (19–87; p=0·016) during the entire follow-up period.

**Figure 2:**
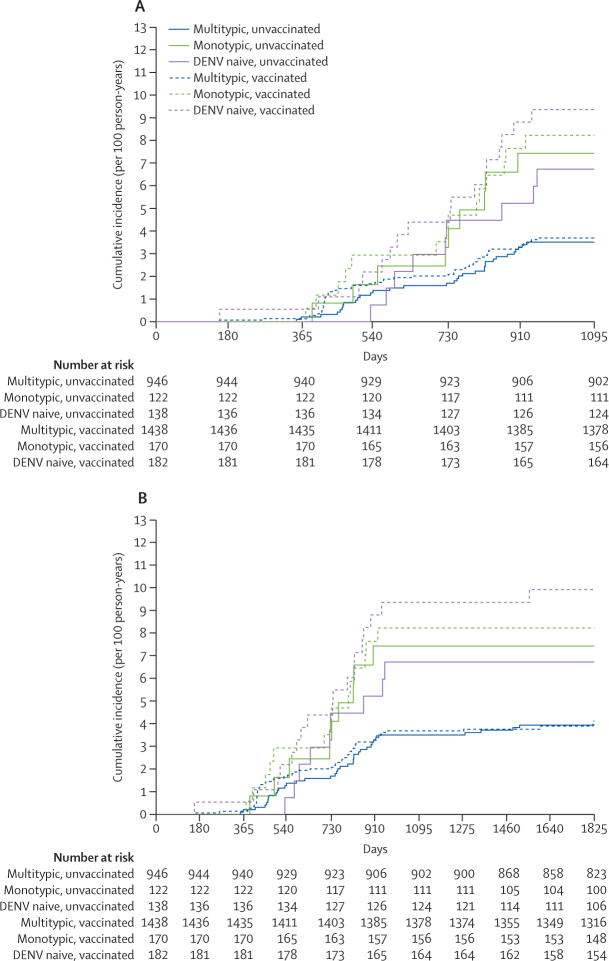
Cumulative incidence rate by baseline serostatus and vaccination status for the first 3 years (A) and entire follow-up (B) Baseline serostatus includes dengue virus naive, monotypic profile, and multitypic profile. COVID-19 restrictions were in place after 3 years. DENV=dengue virus.

We plotted cumulative incidence curves of virologically confirmed dengue from month 0 by baseline serostatus classified as DENV naive, monotypic, and multitypic for the first 3 years and the entire follow-up period (figure 2). The cumulative risk was highest in participants with a DENV naive profile at baseline (vaccinated *vs* unvaccinated; crude HR 1·42 [95% CI 0·63–3·19]; p=0·39 over 3 years and 1·35 [0·62–2·92]; p=0·45 during entire follow-up) followed by participants with a DENV monotypic profile at baseline (vaccinated *vs* unvaccinated; 1·11 [0·48–2·57]; p=0·80 and 1·27 [0·56–2·87]; p=0·57). The HR of participants with DENV multitypic immunity at baseline was similar between vaccinated and unvaccinated participants.

**Figure 3:**
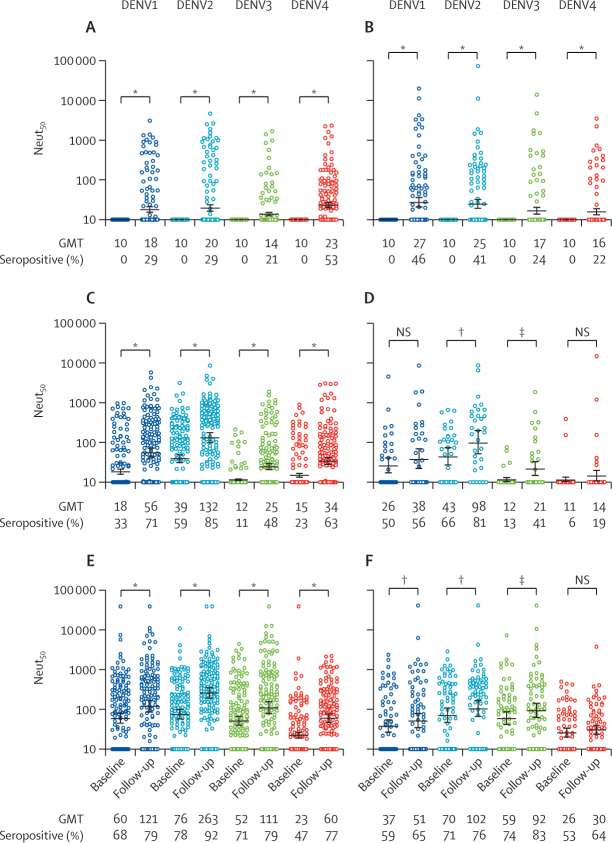
Neut_50_ to DENV1–4 from paired sera at baseline and follow-up, by baseline serostatus and receipt or non-receipt of CYD-TDV Baseline serostatus includes DENV naive (136 vaccinated participants and 93 unvaccinated participants), monotypic profile (142 and 32), and multitypic profile (126 and 66). DENV=dengue virus. GMT=geometric mean titre. Neut_50_=neutralising antibody titres. NS=not significant. *p<0·0005. †p<0·05. ‡p<0·005.

In the first annual blood sample collected 17–28 months after enrolment and vaccination, neutralising antibody titres to all serotypes significantly increased compared with titres at baseline in vaccinated and unvaccinated children with a DENV naive profile (figure 3A, B). After one dose of CYD-TDV, the highest frequency of response in 136 participants was to serotype 4 (72 [52·9%]), whereas only 39 (28·7%), 40 (29·4%), and 29 (21·3%) were seropositive for DENV1, DENV2, and DENV3, respectively. The highest geometric mean titre was to serotype 4 (18, 20, 14, and 23 for DENV1–4, respectively). In the unvaccinated group, the geometric mean titres to DENV1 (27) and DENV2 (25) and seropositivity to DENV1 (43 [46·2%] of 93 participants) and DENV2 (38 [40·8%] of 93 participants) were higher than the geometric mean titres to DENV3 (17) and DENV4 (16) and seropositivity to DENV3 (22 [24%] of 93) and DENV4 (20 [22%] of 93).

Neutralising antibody titres to all four serotypes significantly increased following one dose of CYD-TDV in children with a baseline DENV monotypic profile (figure 3C). In the unvaccinated group at baseline, DENV1 (16 [50%] of 32 participants) and DENV2 (21 [66%] of 32 participants) had the highest percentage seropositivity, whereas DENV2 and DENV3 titres significantly increased in the follow-up samples. Changes were not significant in titres to DENV1 and DENV4 (figure 3D). Geometric mean titres and seroconversion rates were slightly higher in the follow-up samples from vaccinated children than unvaccinated children for DENV1, DENV2, and DENV3, whereas the increases for DENV4 were larger.

Vaccinated and unvaccinated individuals with a baseline multitypic DENV profile had high neutralising antibody titres to all four serotypes at baseline (figures 3E, F). In vaccinated participants, the geometric mean of neutralising antibody titres increased significantly between baseline and follow-up across all four serotypes. Smaller changes in the geometric mean of neutralising antibody titres were observed between baseline and follow-up in unvaccinated children for DENV1, DENV2, and DENV3, and no increase was observed for DENV4.

## Discussion

In this longitudinal, prospective, population-based cohort study, one dose of CYD-TDV did not protect against virologically confirmed dengue in children with a DENV naive or monotypic profile at baseline. Although the number of events was small, a single dose of CYD-TDV was sufficient to confer significant protection against virologically confirmed dengue necessitating hospital admission among those who had a DENV multitypic profile at baseline, but not against any other disease outcomes (virologically confirmed dengue and dengue without or with warning signs).

People with a DENV monotypic profile at baseline were thought to benefit most from CYD-TDV as they are at greatest risk for severe secondary infection, whereas people with a multitypic profile are more likely to already have protective immunity.^8^ However, in the immunogenicity subset of the CYD-TDV phase 3 trial, a slightly lower vaccine efficacy point estimate against virologically confirmed dengue requiring hospital admission was already noted in individuals with a monotypic profile (75·3%, 95% CI 42·7–90·2) versus those with a multitypic profile (81·2%, 21·7–96·8).^18^ A single-dose of CYD-TDV is less immunogenic and likely to be insufficient to induce a secondary-like broad antibody response against DENV1–4 in people with a monotypic profile. Thus, a single dose did not confer protection among participants with a monotypic profile at baseline. An increased risk among vaccinated participants with a monotypic profile at baseline was consistently noted across all categories of virologically confirmed dengue (any, without, and with warning signs) and virologically confirmed dengue needing hospital admission.

We found three reports that assessed a single dose of CYD-TDV. First, a post-hoc analysis of the phase 3 studies found an 80·5% (95% CI 66·2–88·7) protection in participants aged 9 years or older who were seropositive after the first dose for 6 months and null to modest protection in participants who were seronegative.^19^ Second, a hospital-based, case–control study in Cebu showed 48·0% (20·0–66·0) protection against virologically confirmed dengue with warning signs and severe virologically confirmed dengue at 2 years of follow-up.^20^ Third, a case–control study in Parana, Brazil found 25·5% (2·8–42·8) protection against virologically confirmed dengue from a single dose of CYD-TDV in participants aged 19–27 years and 85·8% (58·9–95·1) protection against only DENV4 within 3 years following the mass vaccination.^21^ These studies could not evaluate the vaccine effect in those seropositive at baseline, classified as one versus two or more previous DENV infections.

There was a high incidence rate of virologically confirmed dengue at the site from the beginning of the study to the COVID-19 lockdowns, which started in March, 2020. This high incidence is supported by the immunogenicity analysis in unvaccinated participants with a DENV naive profile that revealed a large increase in neutralising antibody titres between baseline and follow-up samples. In vaccinated children, the neutralising antibodies detected against DENV1, DENV2, and DENV3 probably came from a combination of responses to the vaccine or wild-type DENV infection (or both). The DENV4 serotype of the vaccine was the dominant component, as reported previously.^22^ The immunogenicity results further support the epidemiological findings that even children with multiple previous infections at baseline have virologically confirmed dengue, albeit at a lower incidence rate than those with a DENV naive or monotypic profile at baseline. The vaccine has an additional effect on increasing antibody concentrations across serotypes.

Our study has several limitations. The results are only applicable to receipt of a single dose of CYD-TDV. The study was not a randomised trial, such that factors linked with the decision to receive the vaccine or not could also have been associated with the risk for dengue. The decision to participate in the study was self-determined and might have also affected the decision to subsequently receive the vaccine. Although the overall retention of participants was high, bias remains possible due to the loss of participants or missed events. We integrated several features in the study to assure the validity of the results. We enrolled the participants before the mass dengue vaccination programme, conducted prospective active surveillance in the cohort following standard data and sample collection methods, and confirmed dengue via RT-PCR in an external reference laboratory. Receipt of CYD-TDV documented through linkage with the registry was made without knowledge of the subsequent occurrence of virologically confirmed dengue in participants. Detailed information about potentially confounding variables was collected and controlled for in the analyses. A statistical analysis plan was formulated a priori and carried out by staff in an institution not otherwise involved in enrolment and data collection. During the COVID-19 lockdown, we continued regular telephone contact with the participants and the decrease in febrile episodes and DENV infections is likely to have been a real effect due to social isolation, as also described in other settings.^17^ Due to the large decline in DENV cases, we presented the results during the first 3 years (before the COVID-19 pandemic) and for the whole study.

In summary, we found no protection from a single dose of CYD-TDV among children with a naive or monotypic DENV immune profile at baseline. One dose conferred significant protection against hospitalisation for virologically confirmed dengue among children with a monotypic DENV immune profile at baseline. Since only one dose of CYD-TDV was given, the study cannot be used to inform public health decisions on vaccination but is relevant for children who receive an incomplete regimen. Importantly, the risk of developing virologically confirmed dengue depended on the number of previous DENV infections at baseline and suggests that future dengue vaccine trials should include a more detailed analysis of previous dengue infections (as none, one, or two or more) in participants at baseline.

## Data sharing

De-identified participant epidemiological, clinical, and laboratory data, the study statistical analysis plan, and informed consent forms can be made available according to the University of the Philippines Manila, the University of North Carolina, and the US National Institutes of Health data sharing policy on request to the corresponding author (JD), starting from the time of publication and for the subsequent 3 years.

## Declaration of interests

MY, MVC, JVD, KAA, AMC, and AKS report receiving salaries from 2017 onwards as part of an ongoing separate study (effectiveness of the tetravalent dengue vaccine, CYD-TDV [Dengvaxia] in the Philippines) sponsored by the University of the Philippines Manila and funded by Sanofi Pasteur. JD was an unpaid external consultant in the Extended Study Group for dengue vaccine effectiveness evaluation studies in Asia in 2015 convened by Sanofi Pasteur and is an unpaid investigator of an ongoing separate study (effectiveness of the tetravalent dengue vaccine, CYD-TDV [Dengvaxia] in the Philippines) sponsored by the University of the Philippines Manila and funded by Sanofi Pasteur. AMdS is listed as an inventor on pending patent applications filed by the University of North Carolina related to flavivirus diagnostics. All other authors declare no competing interests. This Article reflects the points of view and thoughts of the authors, and the information, conclusions, and recommendations presented are not to be misconstrued as those of the Philippines Department of Health. The material is presented in the spirit of promoting open access and meaningful dialogue for policy, plan, or programme improvement, and the responsibility for its interpretation lies with the reader.
