# Supplementary material for: Effect of single-dose, live, attenuated dengue vaccine in children with or without previous dengue on risk of subsequent, virologically confirmed dengue in Cebu, the Philippines: a longitudinal, prospective, population-based cohort study
Source: Lancet Infect Dis. 2024 Jul;24(7):737–45. doi: 10.1016/S1473-3099(24)00099-9 (PMC11187693; doi:10.1016/S1473-3099(24)00099-9)
Supplement: Supplementary appendix [file mmc1.pdf]

THE LANCET
Infectious Diseases

Supplementary appendix

This appendix formed part of the original submission and has been peer reviewed. We post it as supplied by the authors.

Supplement to: Ylade M, Crisostomo MV, Daag JV, et al. Effect of single-dose, live, attenuated dengue vaccine in children with or without previous dengue on risk of subsequent, virologically confirmed dengue in Cebu, the Philippines: a longitudinal, prospective, population-based cohort study. *Lancet Infect Dis* 2024; published online March 22. https://doi.org/10.1016/S1473-3099(24)00099-9.

Effect of a single-dose of a live attenuated dengue vaccine CYD-TDV administered to children with or without prior dengue on the risk of subsequent virologically confirmed dengue: A long-term prospective cohort study in Cebu, Philippines

Appendix

Contents | Page

Contents	Page
Study protocol	2
GenBank numbers of the mature dengue virions	36
Supplementary Table 1. Characteristics of the participants by baseline dengue serostatus	37
Supplementary Table 2. Dengue virus (DENV) serotypes during first episode of virologically-confirmed dengue	39
Supplementary Table 3. Cumulative incidence rate (IR) of acute febrile illness and virologically confirmed dengue (VCD) in the cohort, by year of observation	40
Supplementary Table 4. Incidence rate (IR) of acute febrile illness and virologically confirmed dengue (VCD) in the cohort, by dengue serostatus at baseline	41

Effect of baseline dengue serostatus among tetravalent dengue vaccine CYD-TDV (Dengvaxia®) recipients on subsequent virologically confirmed dengue in the Philippines

**Principal Investigator**	**Jacqueline L. Deen, MD, MSc**
**Co-Principal Investigator**	**Michelle Ylade, MD, MSc**
**Laboratory Investigator**	**Ava Kristy Sy**
**Collaborating Institutions**	**Institute of Child Health and Human Development, National Institutes of Health, University of the Philippines Manila, Room 112, NIH Building Pedro Gil St. Manila, Philippines**
	**Department of Health**<br>**DOH Compound, Rizal Avenue, Manila**
	**Research Institute for Tropical Medicine, 9002 Research Drive, Filinvest Corporate City Alabang, Muntinlupa City 1781 Philippines**
	**University of North Carolina, Chapel Hill, North Carolina, USA**
**Study Coordinators**	**Ma. Vinna Crisostomo, MD, Jedas Veronica Daag**
**Research Advisory Board**	**Dr. Lorenz von Seidlein (Mahidol Oxford Research Unit, Bangkok, Thailand)**<br>**Dr. In-kyu Yoon (Global Dengue+ Aedes-transmitted diseases Consortium) /International Vaccine Institute, Seoul, Korea)**
**Proposal version**	**4.1**
**Date**	**05 July 2022**

No part of this protocol may be reproduced or transmitted in any form or by any means, electronic or mechanical, including photocopying, recording, or any information storage and retrieval system, without permission in writing from the Institute of Child Health and Human Development (ICHHD).
For information and support services, please contact ICHHD through email: ichhd-nih@post.upm.edu.ph

Table of Contents

1. Study Summary 3

2. Schematic of Cohort Design 5

3. Background 6
   - 3.1. Background Information 6
   - 3.2. Rationale 8
   - 3.3. Re-analysis of the CYD-TDV data 9

4. Detailed Study Design 9
   - 4.1. Study Objectives/Aims and Hypotheses 9
     - 4.1.1. Primary Objective: 9
     - 4.1.2. Secondary Objectives: 9
   - 4.2. Detailed Description of Study Design 9
   - 4.3. Study Outcome measure(s) 10
     - 4.3.1. Outcome Measures 10
     - 4.3.2. Definitions 10
   - 4.4. Detailed Description of Study Population 13
     - 4.4.1. Subject Inclusion Criteria 13
     - 4.4.2. Subject Exclusion Criteria 14

5. Study Procedures 14
   - 5.1. Recruitment Procedures 14
   - 5.2. Randomization and Blinding Procedures 14
   - 5.3. Study Procedures 14
     - 5.3.1. Cohort follow up 15
     - 5.3.2 Sample collection for febrile cohort patients 15
     - 5.3.3 Sample Collection for Annual Bleeding among Cohort Patients 16
   - 5.4. Data Collection Tools 17
     - 5.4.1. Case Report Form 17
     - 5.4.2. Laboratory test for baseline dengue serostatus determination 17
     - 5.4.3. Laboratory tests for virological confirmation of dengue 18
   - 5.5. Quality Assurance/ Quality Control of Data and Collection 18
   - 5.6. Reasons for and Handling of Withdrawals 19
   - 5.7. Termination of Study 19

6. Protection of Human Subjects/Ethical Considerations 19
   - 6.1. Ethical Standard 19
   - 6.2. Human Subjects Considerations 20
     - 6.2.1. Potential Risks 20
     - 6.2.2. Protections Against Risk 20
     - 6.2.3. Potential Benefits 20
     - 6.2.4. Remuneration 20
     - 6.2.5. Privacy and Confidentiality 21
     - 6.2.6. Vulnerability 21
     - 6.2.7. Recruitment 21

6.3. Informed Consent Process 22
6.4. Safety Oversight Plans 23
6.4.1. Procedures for recording and reporting Adverse Events (AE) and Serious Adverse Events (SAE) 23
7. Statistical Considerations and Data Analysis 23
7.1. Sample Size and Power Calculation(s) 23
7.2. Final Analysis Plan 24
8. Data Dissemination Plan and Knowledge Transfer 25
9. Detailed Timeline 25
10. Archiving 25
11. References 0
12. Annexes 2
Annex A. Data collection forms or other instruments used 2
Annex B. Informed consent and Assent forms 2
Annex C. Terms of reference 2

1. Study Summary

Title	Effect of baseline dengue serostatus among tetravalent dengue vaccine CYD-TDV (Dengvaxia®) recipients on subsequent virologically confirmed dengue in the Philippines
Type of study	Observational cohort study
Study sites	Selected areas of Region 7
Study period	November 2016 to October 2022
Study Duration	5 years observation period, 3 months preparation, 9 months close-out
Study Population	Pupils in selected public schools in Region 7 OR children residents in selected communities who are eligible to participate in the Department of Health (DOH) mass dengue vaccination.
Primary Objective	To determine the relative risk of developing virologically-confirmed dengue (outcome) among Philippine children who received none or at least one dose of dengue vaccine during the DOH mass dengue vaccination, by dengue serostatus at baseline
Secondary Objectives	1. To determine the relative risk of developing severe and/or hospitalized virologically-confirmed dengue among Philippine children who received none or at least one dose of dengue vaccine during the DOH mass dengue vaccination, by dengue serostatus at baseline<br>2. To describe the epidemiologic trends and characteristics of virologically-confirmed dengue, including age, sex, residence, baseline dengue serostatus, previous dengue episode and serotype distribution among school children who received none or at least one dose of dengue vaccine during the DOH mass dengue vaccination.<br>3. To assess the performance of simpler tests such as dried blood spots and serum IgG against the current common standard serum neutralization test for the assessment of dengue seroprevalence at the population level.<br>4. To compare the immune responses of dengue naïve and dengue seropositive individuals who received or did not receive the CYD-TDV vaccine.<br>5. To describe spatial and seasonal distribution of dengue incidence and serotype among children who received none or at least one dose of dengue vaccine during the DOH mass dengue vaccination.<br>6. To evaluate the impact of the COVID-19 pandemic and other disasters on the participants' social, economic, physical, and mental wellbeing.
Trial Designs and Methodology	At least 1,702 eligible children who are residents of selected areas in Region 7 and are eligible to participate in the mass dengue vaccination will be prospectively venipunctured for baseline dengue serologic status. The blood samples will be stored for later serologic testing using neutralization tests and commercial IgG tests. These children will be followed for fever. Children with ≤5 days of fever will be identified and blood drawn for rapid dengue test and RT-PCR. Stored blood drawn at baseline will then be tested for dengue antibodies by neutralization test (all dengue serotypes and possibly other flavivirus antibodies). Febrile acute and convalescent blood samples will be obtained. Annual blood draws to assess subclinical or asymptomatic infections will be performed. In a subset, cellular immune responses will also be assessed.

Primary Endpoint	Virologically-confirmed dengue (by RT-PCR)
Inclusion criteria	• Provide signed informed consent and assent (as applicable)<br>• Be a resident of the selected study areas in Region 7<br>• For school-based immunization: Be enrolled in the 4th grade in a public school during the specified school year for vaccination and aged at least 9 years OR For the community-based immunization: Be a child belonging to the specified age group and resident of the targeted communities of the DOH dengue mass immunization<br>• Be eligible to receive dengue vaccine during the DOH dengue mass immunization in 2017
Exclusion criteria	• Any subject whose parent/guardian refuse to provide informed consent and/or assent<br>• For school-based immunization: Children who are not enrolled in the 4th grade in a public school in Region 7 OR For the community-based immunization: Children who do not belong to the specified age groups and not residents of the targeted communities<br>• Children <9 years old<br>• Children with history of bleeding disorder<br>• Any subject previously enrolled in a dengue vaccine clinical trial
Definitions	• Suspected or probable dengue – any patient on whom the attending physician makes a diagnosis of probable dengue according to the clinical history and the physical examination (without or prior to laboratory confirmation)<br>• Virologically-confirmed dengue (VCD) – a case of suspected or probable dengue with or without warning signs whose serum sample was obtained within 5 days of fever onset and whose result is positive for dengue by real-time reverse-transcriptase polymerase chain reaction (RT-PCR)<br>• Hospitalized VCD – any VCD case admitted to the hospital or stayed in the Emergency Room for treatment for more than 24 hours<br>• Severe dengue – any hospitalized VCD (as defined above) with severe plasma leakage, severe bleeding or severe organ impairment (see definition below)
Duration of follow-up	• 5-years after receipt of 3rd dose of vaccine (subject to availability of funding)

2. Schematic of Cohort Design

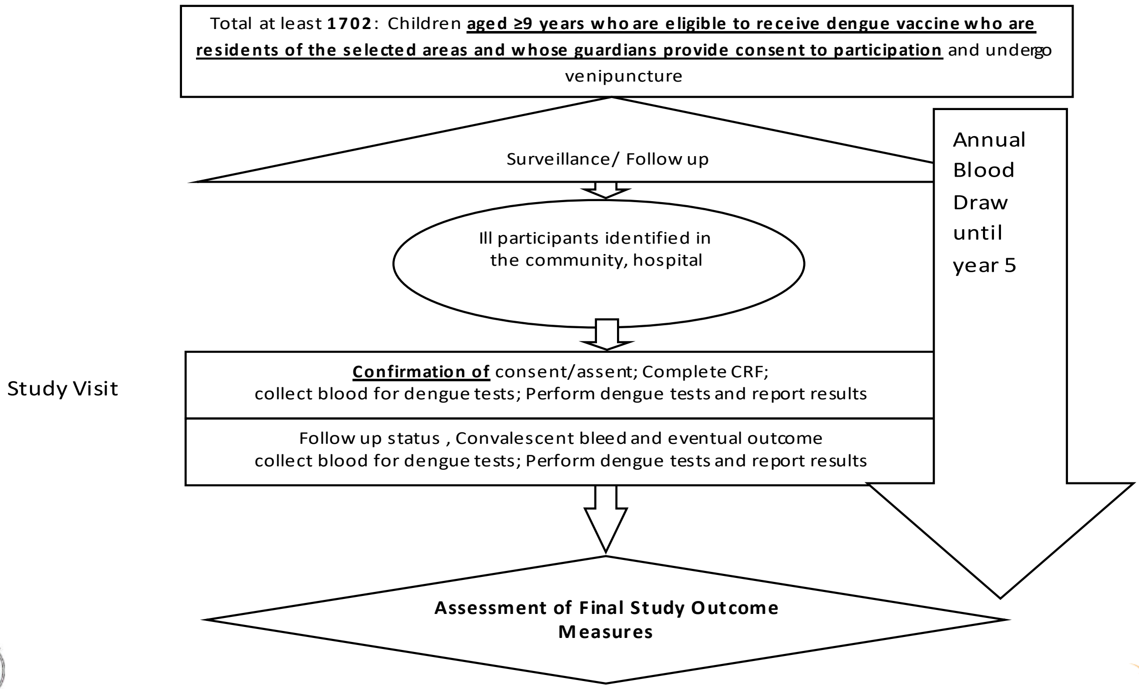

3. Background

3.1. Background Information

Dengue is one of the rapidly spreading mosquito-borne viral disease (1), with global estimates of 58·4 million annual symptomatic dengue infections resulting in about 10 000 deaths per year (2). There are four dengue virus serotypes, DEN-1, DEN-2, DEN-3, and DEN-4, which have considerable antigenic and genetic variation across and within them (3). Cross-neutralization and cross-protection between the four serotypes is limited. Infection with one serotype produces durable, even life-long, homotypic immunity against that same serotype but generates partial and transient cross-protection against the other serotypes, allowing sequential dengue infections in an individual (4). An individual can have up to four dengue infections in a lifetime.

Primary dengue infection is generally asymptomatic or manifests as self-limiting dengue fever, whereas there is a high risk of severe dengue during secondary infection (4). The most prominent feature of severe dengue (previously called dengue hemorrhagic fever and dengue shock syndrome) is a transient increase in vascular permeability resulting in plasma leakage that may lead to circulatory compromise, shock and death (5). Coagulation abnormalities, hepatitis, renal failure, myocarditis or encephalitis may also occur. Although only a small proportion of dengue patients develop severe disease, the lack of definite clinical predictors of progression, the potential to progress to circulatory failure, shock and death, as well as its seasonal outbreaks makes dengue a highly feared illness. Post-secondary infections are associated with a reduced risk of disease (6).

Dengue also causes a substantial economic burden with annual global costs of dengue illness of US$8.9 billion (7). Considering fatal and non-fatal outcomes together, dengue was responsible for 1·14 million (0.73 million–1.98 million) disability-adjusted life-years in 2013 (2) with global estimates of 58.4 million to 96 million symptomatic dengue virus infections (7, 8), including 13,586 fatal cases, 5,838 of which occurred in children (7).

Vector control is the mainstay for dengue disease prevention but is difficult to sustainably apply in low-resource countries and there is little evidence of the effectiveness of any dengue vector control method (9). The first dengue vaccine, CYD-TDV (Dengvaxia™, Sanofi Pasteur), was recently licensed in six countries, the Philippines, Mexico, Brazil, El Salvador, Costa Rica and Paraguay, for use in individuals 9 to 60 years of age living in dengue-endemic areas (10). The basis for licensure was data from two large Phase 3 clinical trials (one among 2-14 year olds in 5 Asian countries and the other among 9-16 year olds in 5 Latin American countries). The pooled vaccine efficacy over 25 months was 60% for all participants, 66% for those 9 years of age or older and 45% for those younger than 9 years of age (11). Baseline dengue serostatus was determined in a subset of trial participants and sub-analysis showed higher rates of vaccine protection among participants who were already seropositive prior to vaccination (i.e. partially dengue-immune), compared to those who were not. The vaccine is not licensed for a younger age group because of a statistically significant increased risk of hospitalization in the third year when the vaccine was given at 2-5 years of age.

In April 2016, the World Health Organization (WHO) Strategic Advisory Group of Experts on immunization recommended that countries consider introduction of the CYD-TDV vaccine in geographic settings (national or subnational) "with high dengue transmission, i.e. seroprevalence of approximately 70% or greater in the age group targeted for vaccination but not below 50%" (12). The basis for the recommendation was a modeling study that used the assumption that CYD-TDV immunologically primes seronegative recipients, causing their first natural dengue infection to be more severe (like secondary dengue infection in unvaccinated individuals) (13). The mathematical model predicts the greatest impact in a high endemicity setting where routine vaccination of 9 year olds at 80% coverage would reduce dengue-related hospitalizations by 13% to 25% over 30 years. In contrast, vaccination in low-transmission

settings with a high population of seronegatives will increase the number of hospitalized dengue cases (14).

Dengue in the Philippines

Among the countries of the Western Pacific Region of the WHO, the Philippines had the highest number of reported dengue cases and deaths due to dengue in 2012 (15). From 2008-2012 there were 585,324 reported dengue cases to the Philippines' Department of Health (DOH) with a case fatality rate of 0.55% (3,195 deaths). Based on comparisons with data from active surveillance, these figures underestimate the true burden of the disease. It is estimated that the actual number of cases may be 7.2 times higher, or a projected 842,867 clinically diagnosed cases of dengue (16). In a prospective cohort study of 1,008 subjects followed for 12 months in Cebu, the incidence of symptomatic and subclinical infections was 1.62 and 7.03 per 100-person-years, respectively (17). In comparison, the incidence of symptomatic virologically confirmed dengue (VCD) and hospitalized VCD cases among controls during the CYD-TDV Phase 3 study in two sites in the Philippines was 6.6 and 0.7 per 100-person years (95% CI, 0.4−1.2), respectively, translating to 10.9% (95% CI, 6.5−16.9) of all VCD cases being hospitalized. About 11% (95% CI, 9.4−12.7) of febrile illnesses among the controls in the Philippine CYD-TDV trials sites were due to dengue (18). These findings support the substantial economic costs of dengue in the Philippines, with an estimated US$345 million in 2012 (18).

No recent seroprevalence studies on dengue have been conducted in the country, but in the CYD-TDV Phase 3 study in the Philippines, dengue serostatus was determined in 602 participants aged 2 to 14 years prior to vaccination (18). (Figure 1) 88.5% of 157 children 9 to 12 years were seropositive at baseline.

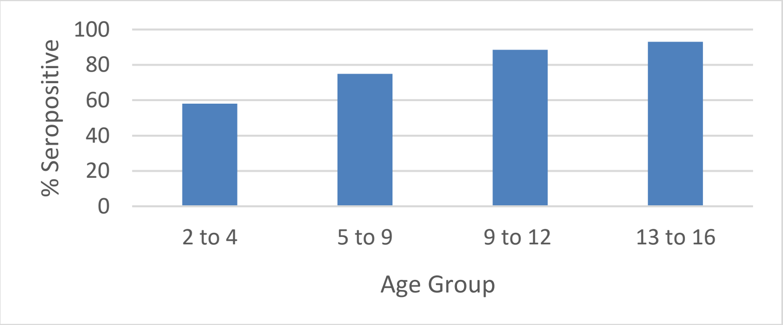

From reference(18)

Figure 1. Dengue seropositivity among 602 study participants in the Phase 3 CYD-TDV study in the Philippines, by age group

School-based Immunization in the Philippines

In 2013, the Philippines' DOH in collaboration with Department of Education (DepEd) and Department of Interior and Local Government (DILG), first introduced the school-based immunization strategy as a means to deliver routine immunizations to school-aged children, to deliver booster doses or to administer new vaccines that are better introduced at a later age. This strategy was piloted in 2013 and was scaled up nationally in 2015. After a successful pilot, the coverage for measles-rubella vaccine reached 60% while tetanus-diphtheria coverage was 73% among Grade 1 pupils.

Considering the substantial health and economic burden of dengue in the country, the Government of the Philippines decided to launch dengue mass immunization in the three administrative regions with the highest disease burden. The Philippines' DOH together with the DepEd and other government agencies, embarked on a school-based dengue immunization of fourth-grade public-school students during the school year 2015-2016. Those eligible for vaccination were public school children who were aged 9 years and older in regions 3, the National Capitol Region (NCR) and 4A. The target number of schools and

fourth-grade students is estimated at: 2,990 and 230,867 in Region 3, 518 and 204,768 in the NCR and 2,728 and 293,533 in Region 4A, respectively. Administration of the first dose was started on 17 March 2016 in Region 3 and on 4 April 2016 in Region 4A and NCR. The first-dose vaccine coverage varied by school (Table 1). The second and third doses are planned to be administered in October 2016 and March/April 2017, respectively. The primary school year in the Philippines is from June to March, thus the fourth graders who receive the first dose of vaccine will be in fifth-grade when they receive the second and third doses. As of 21 July 2016, the number of children who received a dose of the CYD-TDV in the three regions are shown in Table 1.

Table 1. Vaccination coverage in the regions where the school-based immunization was held

Region	Last Day of Vaccination	No. of children targeted for vaccination	No. (%) of Children Vaccinated
**NCR**	July 8, 2016	205,339	101,604 (49.5)
**Region 3**	July 4, 2016	232,660	205,058 (88)
**Region 4A**	July 20, 2016	290,171	182,341 (63)
**TOTAL**		728,170	489,003 (67)

The second and third doses will be administered in the 3 regions in late 2016 and early 2017, respectively. A vaccine effectiveness study is in preparation. This study will assess the protection conferred by the vaccine when given under real life public health settings, the duration of protection and potential herd effects. However, as baseline titres were not obtained prior to the mass vaccination campaign, the study will not be able to assess the impact of baseline serostatus of the vaccinee on the subsequent risk for dengue infection, when the vaccine is given under real life public health settings.

Community-based immunization in the Philippines

In 2013, the Philippines DOH launched a pilot 3-dosed quadrivalent Human Papillomavirus (HPV) vaccination in selected schools in Region 7 and the Cordillera Administrative Region for Grade 5 female pupils aged 10-14 years. In 45 schools in Cebu province targeting 8,123 eligible female pupils, 95.3%, 94.7% and 93.3% received the first, second and third doses, respectively. Following the successful school -based immunization, the DOH then again planned a similar strategy in 2015. However, the 2-dosed quadrivalent Human Papillomavirus (HPV) mass immunization in 20 priority (poorest) provinces in 11 regions of the country, targeting 361,856 females aged 9-10 years was changed to a community-based strategy following concerns by the Department of Education. The community-based HPV vaccination, was able to attain 81% first dose vaccine coverage and 68% second dose vaccine coverage in all provinces combined.

3.1 Rationale

The Philippine government is planning to expand the dengue vaccination to Region 7, using either a school-based or community-based strategy. This provides an opportunity to assess in a large number of participants the effect of baseline serostatus on subsequent risk for dengue.

We propose to compare the effect of seropositive versus seronegative baseline dengue serostatus among Region 7 children recipients of CYD-TDV on their subsequent risk for virologically confirmed dengue. The results of this study will assist policymakers as they decide on how dengue vaccines may be introduced in other areas.

In addition, as the WHO recommends that dengue seroprevalence be used as the important parameter to consider in dengue vaccine introduction, we plan to assess simpler methods in sampling and testing that may be used as potential alternatives to the most common standard neutralization test. Among neutralization tests, the plaque reduction neutralization test (PRNT) is the most widely accepted approach for detecting and measuring dengue antibodies but this is cumbersome, not widely available

and compromised by several limitations, including a wide variation in titer results in response to different testing conditions (19, 20). Simpler serological tests for use at the population level would be more practical but require further validation for this purpose.

This study presents a unique opportunity to assess how the vaccine affects the immune responses of previously immune and non-immune individuals. We are attempting to identify correlates and immune mechanisms responsible for the variable safety and efficacy of CYD-TDV. The previous version of the study protocol was based on analyzing serum antibody responses only. We also need to analyze the B and T cell responses of children because serum antibodies alone provide an incomplete picture. This is especially true for CYD-TDV, which is a dengue-yellow fever chimera that lacks many T cell epitopes present in wild-type dengue viruses. The analysis of cellular immunity is only possible by isolating peripheral blood mononuclear cells (PBMCs) from whole blood.

3.2 Re-analysis of the CYD-TDV data

On November 29, 2018, Sanofi Pasteur, the manufacturers of CYD-TDV recommended a label change in the vaccine. This recommendation was based on recent analysis of data from the CYD-TDV efficacy trials using a newly-developed test (anti-NS1 dengue antibody) that revealed that those who had *not* been exposed to dengue virus prior to vaccination (i.e., dengue-naïve, seronegative) had a twice-higher risk of more severe dengue and hospitalizations compared to unvaccinated participants, regardless of age (21). Subsequently, WHO SAGE recommended CYD-TDV vaccination "only in individuals with a documented past dengue infection either by a diagnostic test or by a documented medical history of past dengue illness". Considering this information, the Philippines' DOH suspended the mass vaccination in December 2017 until further review (22). When WHO issued their guidelines in the clinical development of dengue vaccines in 2012 (23), the information on varying levels of dengue vaccine efficacy and risks among previously dengue immune and non-immune individuals was not yet available. The rapidly evolving dengue and dengue vaccine knowledge base underscores that there is still much to learn in order to understand the mechanisms of dengue protection and risk. Studies to ascertain the immune responses following primary, secondary and post-secondary dengue infections must be performed. Previous studies have shown that primary dengue virus infections result in predominantly serotype-specific polyclonal neutralizing antibody responses and transient heterotypic response (24). However, secondary infections result in more complex mixtures of neutralizing antibodies that recognize serotype-specific and cross-reactive epitopes (25). In the recently concluded WHO consultation, experts recommended that the immunogenicity and efficacy results must be interpreted together with the potential transient heterotypic immunity that could wane over time (26).

In the island of Cebu, the first dose of CYD-TDV was given to ~150,000 children in the mass immunization program. The second and third doses were due to be given in January 2018. Due to the hold order, only the first dose was given.

4 Detailed Study Design

4.1 Study Objectives/Aims and Hypotheses

4.1.1 Primary Objective:

To determine the relative risk of developing virologically-confirmed dengue (outcome) among Philippine children who received none or at least one dose of dengue vaccine during the DOH mass dengue vaccination, by dengue serostatus at baseline

4.1.2 Secondary Objectives:

4.1.2.1 To determine the relative risk of developing severe and/or hospitalized virologically-confirmed dengue among Philippine children who received none or at least one dose of dengue vaccine during the DOH mass dengue vaccination, by dengue serostatus at baseline

4.1.2.2 To describe the epidemiologic trends and characteristics of virologically-confirmed dengue, including age, sex, residence, baseline dengue serostatus, previous dengue episode and serotype distribution among children who received none or at least one dose of dengue vaccine during the DOH mass dengue vaccination.

4.1.2.3 To assess the performance of simpler tests such as dried blood spots and serum IgG against current common standard, serum neutralization test for the assessment of dengue seroprevalence at the population level.

4.1.2.4 To compare the immune response on dengue naïve and dengue seropositive individuals who received or did not receive the CYD-TDV vaccine.

4.1.2.5 **To describe spatial and seasonal distribution of dengue incidence and serotype among children who received none or at least one dose of dengue vaccine during the DOH mass dengue vaccination.**

4.1.2.6 **To evaluate the impact of the COVID-19 pandemic and other disasters on the participants' social, economic, physical, and mental wellbeing.**

4.2 Detailed Description of Study Design

To address the study objectives, we will conduct a prospective, observational cohort study integrating into the Region 7 school-based and/or community-based DOH dengue vaccination.

Immediately prior to receipt of the vaccine, informed consent and assent will be obtained and blood will be collected. The blood samples will be stored for dengue serologic testing using neutralization test and commercial IgG kits. Dried blood spots will also be collected.

In the school-based immunization, fourth graders in public schools of the study area will be followed prospectively for absenteeism and assessed for possible dengue fever. This design would take advantage of the: 1) meticulous, digitized list of the students targeted for dengue immunization that is being compiled by the DOH with a record of CYD-TDV doses received and 2) the real-time daily recording of absences taken at the public schools.

In the community-based immunization, detailed information on the contact information from eligible children participants and their parents/guardians will be obtained. Explicit instructions will be provided on how to contact study staff if the study participants develop fever. Study staff will also check daily with rural health and referral hospitals staff to ensure that children participants with suspected dengue are captured in the study.

In the primary cohort study, we will compare the incidence of dengue fever among vaccine recipients (one, two and three doses), by serologic status at baseline. The assessment of the relative risk of dengue infection in this cohort of children will be crucial as it will inform future use and indications of the vaccine.

The cohort study will evaluate (in a larger number of participants than in the subset of the Phase 3 clinical trials and when the vaccine is given under real public health conditions) whether previously

dengue-unexposed CYD-TDV recipients will develop more severe episodes of virologically-confirmed dengue than those who are already seropositive at the time of vaccination. The challenges of a cohort study include the requirement for more logistic and financial support and the inability to detect rarer outcomes (severe/hospitalised dengue), unless a large number of children are included. Furthermore, since the health care system in the Philippines is primarily fee-for-service and families can choose to access health care anywhere, careful tracking is needed.

4.3 Study Outcome measure(s)

4.3.1 Outcome Measures

Virological confirmation will be primarily by real-time RT-PCR. (The performance of rapid test using RT-PCR as the gold standard will be analyzed separately). Ascertainment of vaccination will be done using the DOH computerized school-based records (for school-based vaccination). For community-based vaccination, master-lists of selected communities on the vaccination information will be obtained from the health centers. Testing of sera for baseline dengue antibodies by neutralization test and using commercial IgG kits will be done later.

4.3.2 Definitions

The following definitions will be used for the study (Figure 1):

- **Suspected or probable dengue** – any patient on whom the attending physician makes a diagnosis of probable dengue according to the clinical history and the physical examination (without or prior to laboratory confirmation)
- **Virologically-confirmed dengue** (VCD) – a case of suspected or probable dengue with or without warning signs whose serum sample was obtained within 5 days of fever onset and whose result is positive for dengue by real-time reverse-transcriptase polymerase chain reaction (RT-PCR)
- **Hospitalized VCD** – any VCD case admitted to the hospital or stayed in the Emergency Room for treatment for more than 24 hours
- **Severe dengue** – any hospitalized VCD (as defined above) with severe plasma leakage, severe bleeding or severe organ impairment (see definition below)

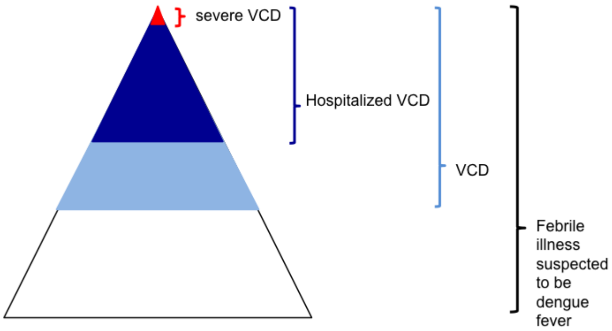

Figure 2: Schematic diagram of febrile illness suspected to be dengue fever

The following definitions, based on the WHO 2009 Dengue Guidelines **(5)** and the DOH PIDSR will be used for classification.

<u>Dengue without Warning Signs</u>

Probable dengue:

Lives in or travels to dengue-endemic area, *with fever, plus any two* of the following:

Headache	Nausea
Body malaise	Vomiting
Myalgia	Diarrhea
Arthralgia	Flushed skin

Retro-orbital pain
Anorexia
AND
•Laboratory test, at least CBC (leukopenia with or without thrombocytopenia). The guideline also state “and/or dengue NS1 antigen test or dengue IgM antibody test (optional)”, which will not be included in the study.

Rash (petechial, Hermann’s sign)
Tourniquet test positive

Confirmed dengue:
•Viral culture isolation
•PCR

Dengue with Warning Signs
Lives in or travels to dengue-endemic area, *with fever lasting for 2-7 days*, *plus any one* of the following:

Abdominal pain or tenderness
Persistent vomiting
Clinical signs of fluid accumulation
Mucosal bleeding
Lethargy, restlessness
Liver enlargement
Decreased or no urine output within 6 hours**
Laboratory: increase in Hct and/or decreasing platelet count

Confirmed dengue:
Viral culture isolation
PCR

Severe Dengue
Lives in or travels to a dengue-endemic area with *fever of 2–7 days and any of the above clinical manifestations for dengue with or without warning signs, plus any of the following*:
- Severe plasma leakage, leading to:

Shock
Fluid accumulation with respiratory distress

- Severe bleeding

- Severe organ impairment

Liver: AST or ALT ≥ 1000
CNS: e.g., seizures, impaired consciousness
Heart: e.g., myocarditis
Kidneys: e.g., renal failure

4.4 Detailed Description of Study Population

The study will be conducted in selected areas of Region 7. (Figure 3).

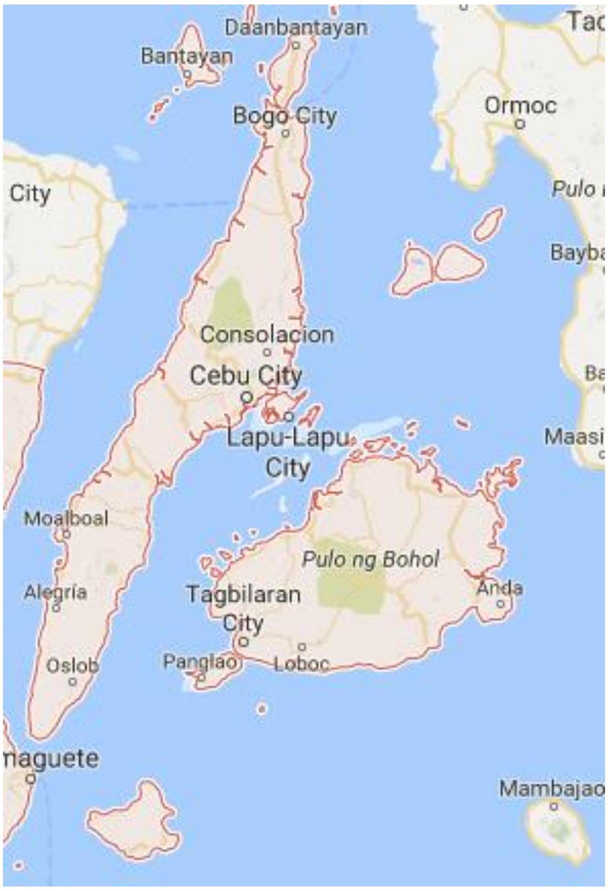

Figure 3: Map of Region 7, including the provinces of Cebu, Bohol and Siquijor

For the study, for the school-based immunization, the population under surveillance will be pupils who are enrolled in the 4th grade in selected public schools in Region 7 during the specified school year and aged 9 years or older and/or children belonging to the specified age groups and residents of targeted areas of the DOH dengue mass vaccination (for community-based immunization).

We aim to recruit at least 1,702 eligible children into the cohort study (see Section 8.1.2 for sample size

Eligibility Criteria

Children eligible to receive CYD-TDV in the DOH dengue mass immunization program will be included in the study. These are children aged 9 years or older who were enrolled in the 4th grade in a public school in Region 7 during the school year 2016- 2017.

4.4.1 Subject Inclusion Criteria

- Provide signed informed consent and assent (as applicable)
- Be a resident of the selected study area in Region 7
- For school-based immunization: Be enrolled in the 4th grade in a public school during the specified school year for dengue vaccination and aged at least 9 years OR
- For the community-based immunization: Be a child belonging to the specified age group and resident of the targeted communities of the DOH dengue mass immunization
- Be eligible to receive dengue vaccine during the DOH dengue mass immunization in 2017

4.4.2 Subject Exclusion Criteria

- Any subject whose parent/guardian refuse to provide informed consent and/or assent
- For school-based immunization: Children who are not enrolled in the 4th grade in a public school in Region 7 OR
  For the community-based immunization: Children who do not belong to the specified age groups and not residents of the targeted communities
- Children <9 years old
- Children with history of bleeding disorder
- Any subject previously enrolled in a dengue vaccine clinical trial

5 Study Procedures

Prior consultations with the National, Regional and Local Offices of DOH and DepEd including local government units (LGU) will be conducted to ensure their participation and commitment. For school-based immunization, meetings with parents and teachers will be conducted to ensure that the community understands the purpose and agree to the conduct of the study. All eligible children will be encouraged to participate.

5.1 Recruitment Procedures

Children from selected areas and/or pupils from public schools located in Region 7 will be included in the study. The area will be chosen for the cohort study based on the proximity to the hospital included in the study and effective coordination between DepEd and DOH in the region. Permissions from the local Department of Education offices as well as school, hospital and LGU officials will be obtained.

For school-based immunization, the study objectives and procedures will be presented during Parent-Teachers Association meetings. A list of the target population (fourth grade public school pupils) compiled by the DOH will be the source document and used as the baseline census.

For community-based immunization, in coordination with the LGU, initial meetings with target participants and their guardians to explain the study procedures will be organized. These meetings will be timed together when the LGUs inform the communities of the DOH mass dengue immunization. Informed consent and assent will be obtained prior to study recruitment. A list of the targeted population will be prepared by the LGUs and will be used as the baseline census.

5.2 Randomization and Blinding Procedures

The study is an observational study; no random assignment to interventions will be performed. The laboratory personnel will be blinded as regards the vaccination status of the subjects.

5.3 Study Procedures

Children aged 9 years or older who are enrolled in the 4th grade in selected public schools during the specified school year and/or residents belonging to the targeted age groups in the selected areas of the DOH dengue mass vaccination are eligible to participate in the study.

Informed consent will be obtained prior to blood draw, just before vaccination. A few drops of blood will be placed on filter paper and the rest of the blood will be processed to obtain sera, stored and later tested for baseline serologic status by neutralization test and using commercial IgG kits (see below). Blood samples on filter paper will be air dried for at least 3 hours, protected by glassine paper and stored in a sealed bag with desiccant at 4°C. Serum samples will be processed as described (see below). Batch shipment of dried blood spots and serum samples to UPM-NIH for storage will be done weekly. Only designated study personnel will have access to the specimens. These will be sent later to a designated laboratory facility following appropriate shipping procedures.

Participants will be under fever surveillance for the duration of the study. Once a participant is confirmed to have fever and is suspected to have dengue, the participant will be asked to confirm their consent and assent prior to continued study procedures as follows:

- The patient and his/her caregiver will be interviewed by trained study personnel who will complete the Case Report Form (CRF). A sticker with a QR or bar code will be attached to the respective patient's CRF.
- Blood will be obtained from each subject for dengue rapid test and RT-PCR. Specimens will be labeled with the subject's initials and subject number and attached to a laboratory request form with an affixed QR or bar code. Dengue rapid tests will be performed in the hospital by trained study staff and the results will be shared with the clinicians managing the case.
- The patient's physician will manage him/her according to hospital/national dengue management guidelines.
- Trained study personnel will follow the case and record his/her course and outcome.
- Stored blood obtained prior to vaccination will be tested using neutralization test for all dengue serotypes (as well as other flaviviruses, if funding allows) later for their baseline serologic status.

5.3.1 Cohort follow up

For the school-based immunization, surveillance for fever will be conducted in the schools. The schoolteachers will be called or sent a text message every other day during school days for pupils who are absent in schools by the study nurses. The fever surveillance is based on absenteeism, which is routinely checked daily in schools. School absence will trigger a telephone call and possibly a home visit, (blood will be obtained for RT-PCR and other tests, if reason for absenteeism is a febrile illness) and followed-up.

For the community-based immunization, guardians will be instructed to contact the study staff directly when the participant has fever. Contact information of the study staff will be provided to all participants and guardians. At the same time, study staff will monitor clinic visits in the health centers and consultations or admissions to the local hospitals.

Informed consent and assent, as appropriate, will be confirmed prior to study procedures when the child has fever. The febrile patient will be advised to visit the nearest RHU or hospital for assessment. The study nurse will go to the health facility where the patient is seen. If he/she is a suspected dengue case as assessed by the physician, the study nurse will collect the required information in the CRF and blood specimen for dengue tests. Results of the dengue rapid test will be provided to the parent/caregiver. The subject will be followed in participating nearby surveillance hospitals included in the area where the vaccination will take place. If the subject is brought to a hospital that is not part of the study, the medical records of the child will be requested from the hospital, after ensuring that the necessary consent has been obtained from the parent/guardian.

5.3.2 Sample collection for febrile cohort patients

Two samples will be obtained from patients who are suspected dengue cases: 1. The first sample will be identified as "acute sample". This will be obtained ideally within the first 5 days of illness as they are likely to be positive during the first 5 days of fever. 2. The second sample will be identified as the "convalescent sample". This follows the recently released DOH Administrative Order (AO) 2018-004 (Interim Guidelines on the Surveillance of Adverse Events among Dengvaxia Vaccinees (AEDV Surveillance). This will be obtained at least 5 days after drawing the acute sample or prior to discharge, whichever comes later.

Blood collection will be done according to the local guidelines. 5 ml will be collected for each sample (i.e. study sample for testing using a rapid dengue test and RT-PCR). Immediately after the blood draw, the

study personnel will affix a label to the tube with the sampling date, subject initials and subject identification number.

For the “acute samples”, blood specimen will be transferred into at least three aliquots. One aliquot will be tested using a rapid dengue test in study hospitals by trained study staff. The second aliquot may be stored at 2° to 8° C and should be shipped to RITM within 5-7 days from sample collection. A third aliquot will be sent to University of North Carolina (UNC) for possible sequencing of DENV virions. A fourth aliquot will be sent to UPM-NIH for storage and IgM/IgG Capture ELISA.

For the “convalescent samples”, specimen will be apportioned into three aliquots. Sera will be initially stored at 2° to 8° C and should be shipped to UPM-NIH within 5-7 days from sample collection. The first aliquot will be tested for IgG/IgM capture ELISA. The second aliquot will be kept at UPM-NIH for storage. The third aliquot will be sent to UNC. In addition to acute RT-PCR and rapid test diagnostics, IgM/IgG serology of sera taken at the acute and convalescent stage provides evidence of seroconversion of immune response following dengue virus infection. This information can corroborate PCR results in addition to providing further information on dengue virus infections that are false negative by PCR testing.

Any leftover specimen for both the acute and convalescent samples will be used for future studies and stored at UPM-NIH for fifteen (15) years after the end of this study. After this period, specimens will be destroyed according to local guidelines. Additional consent will be obtained from the parent for storage and future use of these samples. Only the Principal Investigator and designated study personnel will have access to these specimens.

5.3.3 Sample Collection for Annual Bleeding of the Cohort

Annual bleed from the time of initial collection of baseline blood sample of cohort patients will be conducted over the duration of the study. The first annual bleed will be done at least 6 months from the time of the baseline blood draw. The major focus of this procedure is to define antibody response to the dengue vaccine to identify correlates and mechanisms of protective immunity.

Once informed consent is obtained, approximately 5 mL of blood will be drawn from the child. Blood collection will be done according to the local guidelines. About half of the sample will be processed in the same manner as described above for IgG/IgM capture ELISA and storage, while the remaining blood sample will be sent to UNC for further testing including, but not limited to neutralization tests, depletion assay as previously described (27) and possible transcriptomic and other genetic analysis to further understand the immune response to dengue infection.

During the first annual bleed, at least 100 children (but no more than 300) will be invited for the assessment of B-cell and other cellular immune responses. These children will be bled annually, hence they should have no plans of migrating out of the study areas in the next 4 years. The convenience sample of at least 100 children will be asked to return to the designated phlebotomy areas in the study sites separately during the annual bleed due to the volume of blood that will be drawn. 20 ml is the minimal volume necessary for obtaining sufficient PBMCs for T and B cell studies. Due to the differential effects of baseline dengue serostatus on vaccine efficacy, the children will be chosen based on the results of their baseline dengue serostatus by ELISA. This will include at least 70 baseline seronegative children who are vaccinated, at least 10 baseline seronegative children who are unvaccinated, at least 10 baseline seropositive children who are vaccinated and at least 10 baseline seropositive children who are unvaccinated. Peripheral blood mononuclear cells (PBMCs) will be isolated using density centrifugation techniques as previously described (28). Briefly, 20 ml of blood will be drawn from the children in sodium

heparin tubes. PBMCs will be purified by density gradient centrifugation, resuspended using FBS containing 10% dimethyl sulfoxide, and will be cryo-preserved in liquid nitrogen. Specimens will be tested for T-cell and B-cell specific immune responses as previously described (25, 29-31). PBMC isolation will be performed in a designated facility in Cebu.

5.4 Data Collection Tools

5.4.1 Case Report Form

The following variables will be collected for all with suspected dengue. **The socio-economic and environmental details, important medical events and information on the impact of the COVID-19 pandemic and other disasters will be obtained from all study participants.** Vaccination history will be confirmed against the electronic list of children who received the dengue vaccine in the DOH program.

In addition, the exact location of the participant's house will be mapped using the appropriate mapping application.

Table 4. Variables to be collected

Patient Information	Clinical Information	Laboratory & Vaccination Information	Important Medical Event*	**Impact of the COVID-19 pandemic and other disasters**
• Surveillance site<br>• Date of admission<br>• Name<br>• Address<br>• Mobile/telephone<br>• Age<br>• Date of birth<br>• Gender<br>• 4Ps (NHTS) household<br>• Socioeconomic<br>• Environmental	• Anthropometrics<br>• Number of days with fever<br>• Temperature<br>• Blood Pressure<br>• Excessive/Persistent vomiting<br>• Severe abdominal pain<br>• Mucosal bleeding<br>• Lethargy and restlessness<br>• Petechiae or purpuric rashes<br>• Hematochezia or melena<br>• Difficulty of breathing<br>• Rash<br>• Other physical findings<br>• Laboratory tests: CBC, AST, ALT, creatinine, Chest X-ray<br>• Admitting diagnosis<br>• Final diagnosis	• Date blood specimen collected<br>• Rapid test result<br>• RT-PCR result and genotype<br>• Specimen condition (volume, icepack condition, container type)<br>• Vaccination history<br>• Dates of vaccination	• Important medical event (ie. dog bite, pregnancy, noncommunicable disease, **receipt of any vaccine during the study period** and other conditions)<br>• Date reported<br>• Date of resolution<br>• Disposition<br>• Outcome<br>• Follow-up	• **Questions on financial problems, employment, mental and physical health, and relationships with family members, relatives, neighbors and friend**

	• Disposition (discharged or death)<br>• Length of hospital stay (days)			

*Important medical event is an event that do not fall under the category of a serious adverse event as defined in ICH-GCP or an acute febrile illness but considered significant because of its potential effect on the health status of the participant.

5.4.2 Laboratory test for baseline dengue serostatus determination

Sera will be tested for neutralizing antibodies_using previously described tests (32-34). Briefly, for PRNT, a monolayer of LLC-MK2 kidney cells will be infected with 30–50 plaque-forming units of DENV in the presence of four-fold serial dilutions of heat-inactivated sample on a 12-well plate. For each dilution, the number of virus plaques was counted and compared to the number of plaques in a control where no sample was added. Current reference strains will be used. High throughput neutralization tests (34) validated against the PRNT may also be used following WHO guidelines (32).

Neutralization test is time-consuming and resource intensive, as such, the samples will be stored and tested at a later date. The performance of neutralization test requires experienced laboratory personnel in laboratories with set-up for the test and up to a maximum of 40 specimens may be tested per week. Commercial IgG test kits will be chosen based on recommendations of dengue experts, and will be performed based on manufacturer's recommendations.

To assess the utility of the dried blood spot against neutralization test, dried blood spots will be collected and stored at UPM-NIH. Dried blood spots will be eluted with phosphate buffered saline containing 0.05% Tween 20 and agitated at 300 rpm overnight at 4°C. DBS eluates will be centrifuged to remove debris before the eluates are collected and stored at -20°C until assayed.

5.4.3 Laboratory tests for virological confirmation of dengue

5.4.3.1 Reverse Transcriptase Polymerase Chain Reaction (RT-PCR)

Total nucleic acid will be extracted from the serum samples using the QIAmp Viral RNA kit (QIAGEN, Inc., Valencia, Calif.) kit according to the manufacturer's protocol. The Dengue detection and serotyping of the serum samples will be done using the Simplexa Dengue assay (Focus Diagnostics, Cypress, CA, USA). The assay is a real-time RT-PCR that distinguishes serotypes, using two-reaction mixture, Dengue 1 and 4 in one reaction, and Dengue 2 and 3 in another reaction. Bi-functional Scorpion-based fluorescent probe-primers together with reverse primers will be used in this method to amplify NS5, NS3, NS5, and capsid genes of DENV-1, DENV-2, DENV-3, and DENV-4, respectively. Also, an RNA internal control (RNA IC) will be included to monitor the RNA extraction process and to detect RT-PCR inhibition.

Briefly, two reaction mixes (1 & 4 and 2 & 3) will be prepared according to the manufacturer's instructions. These mixes consist of serotype-specific primers mixes, Taq Polymerase, and RT enzyme. Five microliters of the reaction mixes will be added into designated wells of Universal Disc (3M Focus Diagnostics) followed by the addition of 5 ml RNA samples, Molecular Control (MC, consisted of inactivated dengue virus serotypes -1, -2, -3, and -4), and No Template Control (NTC). Following the sample addition step, wells will be sealed and the disc will be then inserted into the 3M Integrated Cycler real-time RT-PCR instrument (3M-Focus Diagnostics). Samples will run using pre-programmed conditions set by the manufacturer. Data

collection and analysis will be performed using Integrated Cycler Studio Software version 4.2. The criteria for valid detection are as follows: the positive detection of MC, negative detection of NTC, and the presence of RNA IC amplification curve in negative samples. Samples will be reported as positive for DENV infection when the Ct value of each serotype was ≤ 40.0, and ≠ 0.

5.4.3.2 Loop-Mediated Isothermal Amplification (LAMP) Assay

The LAMP Assay is a rapid, highly specific single step nucleic acid amplification and detection technique that has been used in the testing for various pathogens in different specimens (35). Biotek-M^{TM} Dengue Aqua Kit is a miniaturized LAMP assay that detects dengue virus infections. Briefly, extracted RNA is incubated to 63°C in reaction tubes for one hour, followed by incubation at 80°C for two minutes to stop the reaction. SYBR green dye is then added to the reaction mix before viewing using the Viewpoint LED transilluminator. Based on a pilot study of 119 clinical samples, the kit is 85.1% sensitive and 80% specific when the test is done during the first seven days of illness. Sensitivity improves to 92.3% when the test is done during the first 3 days of illness when compared to PCR. Currently there are more than 100 personnel trained to perform the test in 70 hospitals and rural health units. The LAMP test is included in this study to provide immediate preliminary results for clinical use. The performance of LAMP using RT-PCR as the gold standard will be analyzed separately

The Biotek-M^{TM} Dengue Aqua Kit is part of the “Lab-in-a-Mug Project,” wherein all diagnostic kits are integrated and miniaturized in an isothermal unit as small as a “mug” which functions as a multi-infectious disease diagnostic device similar to a portable laboratory. The “Lab-in-a-Mug” was conceptualized, designed, and piloted by Filipino scientists at the Institute of Molecular Biology and Biotechnology, National Institutes of Health, University of the Philippines Manila.

5.4.3.3 NS1 antigen detection

If the LAMP cannot be optimized in the study sites, another rapid test will be used. NS1 antigen detection kits that are locally available (e.g. SD Bioline Dengue NS1 Ag) will be used. The results will be provided to the parents/guardians.

5.4.4 Laboratory tests for PBMCs

From the PBMCs, we will isolate CD4 and CD8 T cells to determine the frequency of cells recognizing specific peptide epitopes on DENV, yellow fever and other related flaviviruses. We will also characterize the functionality of different antigen specific T cell subsets. These results will be compared and contrasted with known T cell responses to natural dengue virus infections and other flavivirus vaccines.

From the PBMCs, we will also immortalize memory B-cells by EBV transformation. The memory B cells will be profiled to determine the frequency and specificity of DENV reactive clones. In particular, we will focus on clones producing antibodies directed to prM, E and NS1 antigens. Antigen specific B cells will be further characterized to identify clones producing functionally neutralizing antibodies. The B cell and antibody repertoire in vaccinated children will be compared to the known repertoire in individuals who have been naturally infected DENVs or vaccinated with attenuated flaviviruses.

5.5 Geospatial mapping

Household locations will be mapped and recorded.

5.5 Quality Assurance/ Quality Control of Data and Collection

Study coordinators and nurses will be responsible for the completeness, validity, consistency, timeliness and accuracy of study data. The study coordinators will create source document templates for the study that are designed to ensure that there is thorough documentation of all procedures required by the protocol at each study visit. Study coordinators and nurses record data on these source documents in accordance with the internal standard operating procedures. Paper case-patient report forms will be stored in secure, locked cabinets to which only staff have access. Original CRFs will be initially kept on-site. At least once per month, the overall project coordinator will visit the surveillance site and review all completed forms. Forms that have missing data will be reviewed to determine the reason why the data is missing. If the coordinator determines that it is possible to recover the data, the site nurses will attempt to obtain the missing information. All reviewed, completed CRFs will be transported to a central data processing center in the ICHHD and stored in a secure, locked cabinet.

At the central data processing centers in the sites, all data will be entered into an electronic database. Records from the evaluation will be stored in the Institute of Child Health and Human Development (ICHHD); duplicate copies will be stored in the Hospital Surveillance office. Original records in ICHHD will be kept for 5 years after evaluation completion then destroyed. Duplicate records in the hospital will be destroyed after evaluation closure and all originals have been transferred to ICHHD.

Coordinators will also be responsible for recording data on the electronic data capture system that will be developed for the study. The study coordinators and the data manager are responsible for 100% quality control (QC) of source documents at each site. QC involves checking for completeness and for compliance with source documentation standards as well as crosschecking data in the data entry program and the source documents to ensure accuracy and consistency. The data management staff is responsible for ensuring that the data are stored and the website is functioning. Data from the study will be available online and maybe viewed by the study coordinators and investigators remote from the study site. The website will be username and password protected, both of which are assigned by the administrator to responsible study staff. Data on the website will be reviewed regularly to ensure that the data entered is correct. The website will be maintained by specific study staff who continually monitors the integrity of the system and the server. Data managers must participate in project management meetings. At each site the study nurse is designated and trained to serve as back up to the data manager.

Furthermore, the monitor will assess the compliance of the local staff by cross-checking the data entered in the forms, the program and the laboratory entries. Data cleaning will be regularly performed by the Data Management staff.

5.6 Reasons for and Handling of Withdrawals

All subjects who withdraw consent will be discontinued from participating. As much as possible all study subjects will be followed until outcome is obtained. The study nurse will follow up all patients until an outcome is obtained, if the subject is hospitalized.

5.7 Termination of Study

The following are criteria for possible premature study discontinuation:

- At the discretion of the, UPM Research Ethics Board, investigators (see halting rules below)
- Request by the participant to withdraw.

6 Protection of Human Subjects/Ethical Considerations

6.1 Ethical Standard

The safety of research participants is foremost. The study will be conducted according to the 2013 Declaration of Helsinki and all local rules and regulations. The study will be submitted to the UPM Research Ethics Board (UPM-REB) for ethical approval. Additional ethical approvals may be required by participating hospitals. The study will comply with the Data Privacy Act of 2012.

6.2 Human Subjects Considerations

6.2.1 Potential Risks

Potential risks to the participants are minimal and may involve risk during blood draw and potential breach of data privacy. Prior to vaccination, blood will be drawn from potential vaccinees. This will be used to assess their baseline dengue serologic status. Febrile subjects will only have additional blood drawn for acute and convalescent sample, annual and PBMC bleed. The venipuncture procedure may cause temporary pain, anxiety and discomfort to the subject. The puncture sites may lead to local swelling and tenderness. The site may also rarely be a source of infection. These risks will be minimized as only trained personnel observing standard aseptic techniques will perform the procedure. Data that will be collected from the subjects will include information for dengue suspects.

6.2.2 Protections Against Risk

The study, including all study procedures and its potential risks and benefits, will be explained to the subject and the subject's parent or guardian through the informed consent process. The study procedures will only be performed after informed consent/assent is obtained.

All the necessary guidelines in compliance with the UPM-REB procedures and guidelines will be followed to protect patients if they are entered into the study. The privacy of patients will be respected and confidentiality of data will be maintained. Only relevant study staff will have access to subject data during the study period. Patients found to have other illnesses aside from dengue will be referred to health authorities for management.

Data from the parents and the subject will be collected by study personnel. They will be trained to collect information in a respectful and sensitive manner. **Information about the patient's household location will be collected using geospatial mapping. In the event of breach of privacy, there is a potential risk that the exact location may be identified or the individual is re-identified using the mapping coordinates. In order to avoid this, we will ensure that all information will be de-identified and we will utilize approaches to mask the participant's identity.** We will follow strict eligibility criteria for all study patients, and will not perform or repeat unnecessary testing. Prior to the blood draw, the procedure will be explained to parents and to the child so that they know how the procedure will be performed. All blood draw procedures will only be performed by trained personnel following strict standard techniques.

Access to the specimens collected in this study will be limited to staff involved in the study. Specimens collected during the study will be stored and used in the future for other tests. Permission from the parents and study participants will be obtained during the informed consent process, before the specimens will be stored for later use for other tests not included in the study. Ethical approval is required for use of specimens in other studies and will be obtained from UPM-REB if specimens will be used for this purpose.

6.2.3 Potential Benefits

In the cohort study, subjects from the cohort who have febrile illness will have free dengue diagnostics tests (LAMP as above).

6.2.4 Remuneration

The subjects will receive some remuneration for her/his participation. **The subject will also receive Php 500 during the acute blood collection and Php 500 during the convalescent blood collection.** Subjects will be given a small token during the baseline blood draw. They will not incur any additional cost to participate in the study. In the cohort study during fever surveillance, if study procedures (blood draw for dengue rapid tests and RT-PCR) will be done in the nearest healthcare facility (health center or hospital), all travel costs incurred by the subjects will be paid by the study. During the annual bleed, a renumeration of ~~Php300~~ **Php 500** will be given to all cohort participants and their travel costs will be reimbursed by the study. For the subset of children for PBMC blood draw, since this will require additional visits, additional informed consent for the blood draw will be obtained. A remuneration of Php1,000 will be provided to each child who will participate in the PBMC blood draw.

The study will not pay for the hospitalization or any diagnostic or therapeutic expenses incurred during hospitalization other than the diagnostic tests for dengue (rapid test and RT-PCR). However, if there are tests (such as AST, ALT and creatinine) requested by the attending physician and this will assist the study team in assessing if the patient is a severe dengue case, that the patient cannot pay or the hospital does not provide for free, these tests may be paid by the study. All diagnostic and therapeutic interventions will be decided upon by their healthcare provider based on the prevailing standard of care.

6.2.5 Privacy, Confidentiality and Data Protection Plan

Every effort will be made to maintain the confidentiality of all data collected. Case report forms and data files will be kept in secured areas at the study site. Physical access to these areas will be restricted to only those involved with the study. **The study will be conducted according to the Implementing Rules and Regulations of the Data Privacy Act of the Philippines, respecting the fundamental human rights of privacy and National Ethical Guidelines for Health and Health-Related Research.**

Each study participant is assigned a unique study number. A document that contains their coded study number and names will be kept in a separate file. Their names will not appear in the data bases that are shared between staff such as those for validation and data analysis.

Information on geospatial mapping will also be de-identified and we will make every effort to maintain the confidentiality of information collected. In data reporting, we will use different approaches in masking geographic identifiers to be able to limit individual reidentification.

Information will be stored in password-protected files that can only be accessed by assigned study personnel. Electronically transmitted data will be secured as described above. Clinic forms that contain personal identifiers (the patient's name, address, phone numbers and other identifiers that will permit study personnel to perform a phone call or a home visit for those included in the cohort study) will be maintained at the sites in secured file cabinets to which only study personnel have access. Only authorized personnel will have permission and ability to access the data. The patient's case report form will be labeled only with the patient's study number.

Access to the computer data files will be restricted. Those authorized to examine the databases will be supplied with passwords for access. At the end of the study, paper forms (without personal identifiers) will be filed and stored in locked cabinets in a secure location in UPM-NIH. The electronic files will be stored in discs (DVDs) that will be stored in a secure location at UPM-NIH together with the paper forms.

6.2.6 Vulnerability

The study population belongs to a vulnerable group. All measures will be observed to protect their rights. Before any study procedures, an informed consent and assent (as applicable) will be obtained. Measures will be instituted to minimize possible risks from any study procedure, in particular blood draw. The privacy and confidentiality of their data will be protected. It will also be emphasized to the potential

subjects and their parents or guardians that participation is voluntary and their decision to participate or not will not in any way affect the services they get from their respective healthcare providers and from their schools.

6.2.7 Recruitment

For the cohort study, the population under surveillance for the school-based immunization will be children aged 9 years or older who are enrolled in the 4th grade in public schools during the specified school year and/or for the community-based immunization, children in the specified age group in targeted areas for the DOH dengue mass immunization. The cohort will consist of children who are residing in selected areas of Region 7. The site was chosen based on the number of eligible population, its accessibility to the hospital included in the study.

All eligible children will be invited to join the study during one of the PTA meetings of the schools or during one of the meetings organized by the LGUs. We will inform them of the need for written consent for all potential subjects to participate in the baseline blood draw and prospective surveillance. Furthermore, for the school-based surveillance, absenteeism is regularly checked in schools. Only children who will be absent in schools for more than 2 days will be contacted by a study nurse to ask if they have fever. Pupils in selected schools will be under fever surveillance during the study period and those who fulfill the criteria for suspected dengue will be invited to participate and undergo further study procedures. Written informed consent and assent will be obtained at this time.

6.3 Informed Consent Process

An overview of the study including the purpose, the inclusion criteria, the study procedures and the voluntary nature of participation will be presented during Parent-Teachers Association (PTA) meetings and during the meetings organized by the LGUs for vaccination. We will inform parents and guardians about the baseline blood draw followed by a fever surveillance. For school-based immunization, this surveillance is anchored in the checking of pupils' attendance by teachers and that during the study period they may receive a phone call or home visit when their son or daughter is absent from school. Emphasis will be placed on why the baseline blood draw and surveillance are important and what questions will be asked during these calls or home visits. The list of the target population (fourth grade public school students) compiled by the DOH will be the source document and used as the baseline census. For the community-based surveillance, parents and guardians will be asked to contact the study staff if the child participant has fever of more than 2 days and proceed to the local health center or the local hospital, as necessary. At the same time, study staff will monitor consultations in the health centers and hospitals for fever.

Written informed consent will be obtained from one of the parents or from the guardian of the eligible child in the study. In addition, following the National Ethical Guidelines, documentation of verbal assent will be done for eligible children aged 7 to less than 12 years of age. A written simplified assent will be obtained from eligible children aged 12 years to less than 15 years of age. For eligible children/adolescents aged 15 years to less than 18 years of age, the potential participant will co-sign the informed consent form signed by the parent/guardian (36). Prior to their use, the informed consent and assent forms will have a written approval from the University of the Philippine Manila-Research Ethics Board (UPM-REB). Please refer to the informed consent and assent forms (Annex C).

The informed consent and assent process will be performed in the school where the eligible child will receive the vaccine.

Eligible children and parents/guardian will be given the approved informed consent form (ICF) and assent form to read. The ICF and assent forms will be written in the language understandable to the subject and/or the parent/guardian. The study staff of the site will explain to them the study as well as the procedures included in the study. The explanation will emphasize the following:

- An overview of the study including the purpose of the study
- The procedures the subject has to undergo as part of the study. This includes the blood draw prior to the receipt of the vaccine.
- The voluntary nature of the study emphasizing that they can withdraw for whatever reason anytime during the study without fear of this decision affecting the standard medical care received from the health centre or the services they get from their schools. They can also still receive the vaccine even if they do not have their blood drawn.
- Confidentiality will be observed throughout the study. Only study staff will be aware of their identities and their respective data.

The potential subject and his/her parent or guardian will be given time and opportunity to ask questions and be given answers to their satisfaction. If they agree to be part of the study, they will sign and date two ICFs and if applicable two assent forms. One form will be kept by the study team and the other will be kept by the subject or parent. All study procedures will only be performed after the signing of the ICF and if applicable, the assent form. If they refuse to be part of the study, the vaccine will still be given.

The following are special circumstances in obtaining ICF.

- Illiterate parents/guardians of eligible children should have an impartial literate witness who will affirm through his/her signature that the parent/guardian of a potential subject gave his/her informed consent. The illiterate parent/guardian of the subject should affix his/her thumbprint in the ICF as well.
- For parents of potential subjects with cognitive impairment, the guardian of the child will have to sign the informed consent for him/her.

During the fever surveillance, once a child from the cohort is ascertained to have febrile illness, a study staff will make a home visit. During the home visit, informed consent will be confirmed prior to any further study procedure, including obtaining data. An additional informed consent will be obtained

The procedures for the informed consent process are the same as those described in Section 6.3.

6.4 Safety Oversight Plans

The study will assess the impact of baseline dengue serologic status on subsequent dengue infection after receipt of CYD-TDV, which is approved by the Philippines FDA for commercial use. This is an observational study that will not require a Data Safety Monitoring Board.

During the fever surveillance for the cohort study, we will refer, when appropriate, any suspected dengue cases to the corresponding health center or hospital. We will also report any observed Adverse Events Following Immunization (AEFI) through the Philippine Integrated Disease Surveillance and Response (PIDSR) system, when applicable.

6.4.1 Procedures for recording and reporting Adverse Events (AE) and Serious Adverse Events (SAE)

No medications or biological agents will be given to the subjects during the study. However, we will be following up children who are eligible to receive a commercially available dengue vaccine from a government program.

All febrile illnesses among the children will be followed. Since children will be followed following dengue vaccination, such febrile illness may qualify as an AEFI. As such, the study staff will report the event, following the AEFI surveillance of the government. The staff will refer the child to the nearest health center or hospital for standard health care.

The study will draw blood for rapid dengue test and RT-PCR. Possible risks from the blood draw have been discussed in Section 6.2.2. Potential Risks. Children will be observed for 30 minutes after the procedure to document any adverse effects. Any adverse event following a blood draw procedure will be recorded in the case report form. All adverse events resulting from the blood draw will be given standard medical care either from the study staff (nurse) or from healthcare staff of the school, the hospital or health center where the procedure was done. Follow up of those who have adverse events will be conducted for the next 24 and then again at 72 hours, until resolution of symptoms.

7 Statistical Considerations and Data Analysis

7.1 Sample Size and Power Calculation(s)

The relative risk for symptomatic dengue among seronegatives compared to seropositives is unknown. Hadinegoro et al (11) showed that the relative risk among seronegatives for hospitalized dengue for children aged 9 years and older ranged from 3 to 3.7. To assess the impact of baseline dengue serologic status among recipients of CYD-TDV against symptomatic virologically-confirmed dengue among children, sample size is driven by the proportion of dengue seropositives at baseline (70%), proportion of seronegatives who develop virologically-confirmed dengue, relative risk of any virologically-confirmed dengue of at least 3.5 and 20% loss to follow-up or non-participation. We will attempt to continue follow-up of school dropouts, but this may be significant in this cohort since it is estimated that in 2013 ~6% of 5-15 year old children in Region were out of school (37), we adjusted an additional 6% for possible school dropouts until senior high school, and with 80% power and alpha of 0.05, we would need to follow at least 1,702 pupils (Table 3).

Table 3. Sample size requirements based on varying dengue seronegative participants and risks of dengue, assuming 80% power, alpha is 0.05

% Seronegative	Risk	Sample size
20	3	3239
25	3	2671
30	3	2299
20	3.5	2412
25	3.5	1981
30	3.5	1702

7.2 Final Analysis Plan

Figure 3 summarizes the plan for analysis. Demographic, environmental and socioeconomic variables will be compared between seropositive and seronegative at baseline vaccine recipients with both bivariate analysis and multivariable regression models.

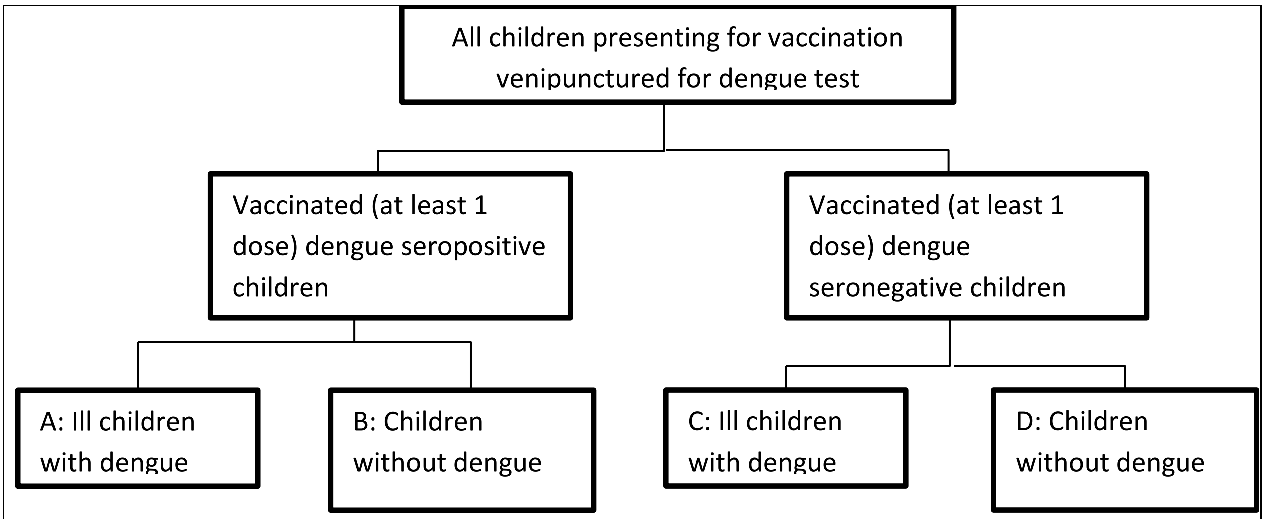

$I_{sero+} = \frac{A}{A+B}$ $I_{sero-} = \frac{C}{C+D}$ $RR = \frac{I_{sero-}}{I_{sero+}}$

where:

- I = incidence
- sero+ = participants seropositive at baseline
- sero- = participants seronegative at baseline
- RR = relative risk

Figure 4. Plan for analysis

8 Data Dissemination Plan and Knowledge Transfer

The results of the study will be presented to policymakers, physicians and other health care professionals through meetings with stakeholders, conference presentations and publications in reputable journals. Since the Department of Health is a collaborator, the results from the study will be used to support guidelines and policy development.

The data generated as well as all specimens obtained in the course of the study will belong to the investigators. The Principal Investigator will ensure that all investigators have access to draft publications and presentations prior to finalization. All personal identifiers will be removed prior to transporting data for analysis.

9 Detailed Timeline

Please see next page for the updated Gantt chart of the study.

10 Archiving

All study documents will be stored in UPM-NIH until the end of the study. Temporary storage in locked cabinets will be made available in secure sites in or near the local hospitals for each site prior to transport to UPM-NIH. Study documentation includes all CRF, workbooks, laboratory reports, source documents, monitoring logs, investigators correspondence, Ethics Committee / regulatory documents (e.g., confidentiality agreement, signed protocol and amendments; specimen list, shipping documents, etc.), and any other reports or records of procedures performed in accordance with the protocol and study-specific Standard Operating Procedures (SOP).

In addition, the study will comply with specific local regulations/recommendations as regards patient record retention after completion. The original recording of study-related observations will be retained as the source document for 5 years.

"Effect of baseline dengue serostatus among tetravalent dengue vaccine CYD-TDV (Dengvaxia®) recipients on subsequent virologically confirmed dengue in the Philippines"

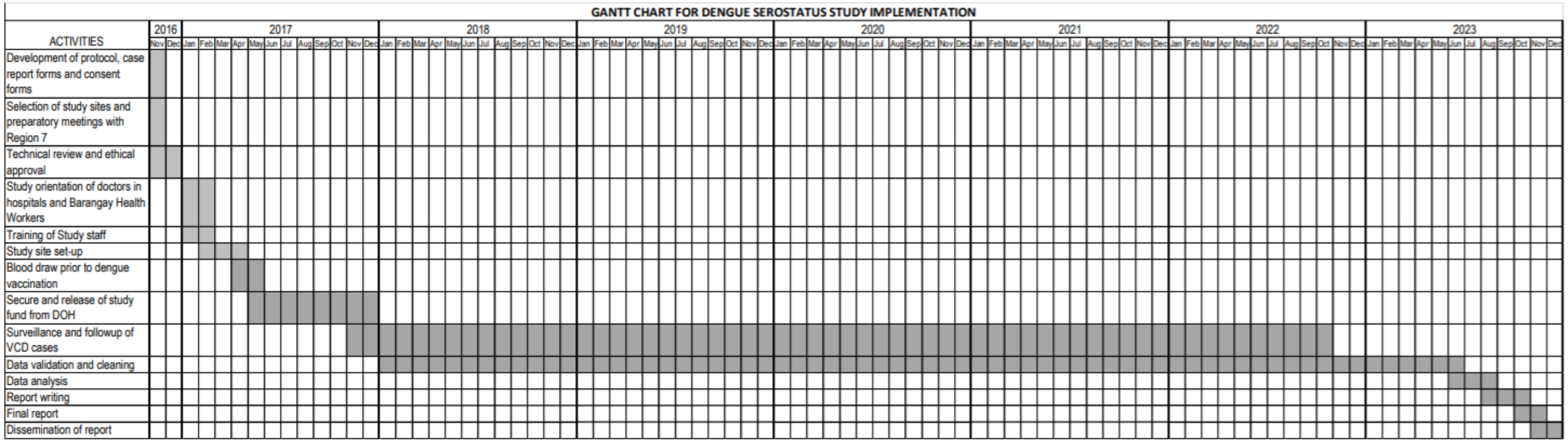

11 References

1. Messina JP, Brady OJ, Scott TW, Zou C, Pigott DM, Duda KA, et al. Global spread of dengue virus types: mapping the 70 year history. Trends Microbiol. 2014;22(3):138-46.
2. Stanaway JD, Shepard DS, Undurraga EA, Halasa YA, Coffeng LE, Brady OJ, et al. The global burden of dengue: an analysis from the Global Burden of Disease Study 2013. The Lancet Infectious diseases. 2016.
3. Heinz FX, Stiasny K. Flaviviruses and flavivirus vaccines. Vaccine. 2012;30(29):4301-6.
4. Simmons CP, Farrar JJ, Nguyen v V, Wills B. Dengue. The New England journal of medicine. 2012;366(15):1423-32.
5. WHO. Dengue: Guidelines for Diagnosis, Treatment, Prevention and Control: New Edition. Geneva2009.
6. Olkowski S, Forshey BM, Morrison AC, Rocha C, Vilcarromero S, Halsey ES, et al. Reduced risk of disease during postsecondary dengue virus infections. J Infect Dis. 2013;208(6):1026-33.
7. Shepard DS, Undurraga EA, Halasa YA, Stanaway JD. The global economic burden of dengue: a systematic analysis. The Lancet Infectious diseases. 2016.
8. Bhatt S, Gething PW, Brady OJ, Messina JP, Farlow AW, Moyes CL, et al. The global distribution and burden of dengue. Nature. 2013;496(7446):504-7.
9. Bowman LR, Donegan S, McCall PJ. Is Dengue Vector Control Deficient in Effectiveness or Evidence?: Systematic Review and Meta-analysis. PLoS Negl Trop Dis. 2016;10(3):e0004551.
10. Pang T. SAGE committee advice on dengue vaccine. The Lancet Infectious diseases. 2016;16(8):880-2.
11. Hadinegoro SR, Arredondo-Garcia JL, Capeding MR, Deseda C, Chotpitayasunondh T, Dietze R, et al. Efficacy and Long-Term Safety of a Dengue Vaccine in Regions of Endemic Disease. The New England journal of medicine. 2015;373(13):1195-206.
12. WHO. Summary of the April 2016 meeting of the Strategic Advisory Group of Experts on immunization (SAGE) http://www.who.int/imunization/sage/en/index.html, accessed April 2016. 2016.
13. Flasche S, al e. The long term safety, public health impact, and cost effectiveness of routine vaccination with Dengvaxia®: a model comparison study. PLoS Med. 2016.
14. Ferguson NM, Rodriguez-Barraquer I, Dorigatti I, Mier YT-RL, Laydon DJ, Cummings DA. Benefits and risks of the Sanofi-Pasteur dengue vaccine: Modeling optimal deployment. Science. 2016;353(6303):1033-6.
15. Arima Y, Chiew M, Matsui T, Emerging Disease S, Response Team DoHS, Emergencies WHOROftWP. Epidemiological update on the dengue situation in the Western Pacific Region, 2012. Western Pac Surveill Response J. 2015;6(2):82-9.
16. Edillo FE, Halasa YA, Largo FM, Erasmo JN, Amoin NB, Alera MT, et al. Economic cost and burden of dengue in the Philippines. Am J Trop Med Hyg. 2015;92(2):360-6.
17. Alera MT, Srikiatkhachorn A, Velasco JM, Tac-An IA, Lago CB, Clapham HE, et al. Incidence of Dengue Virus Infection in Adults and Children in a Prospective Longitudinal Cohort in the Philippines. PLoS Negl Trop Dis. 2016;10(2):e0004337.
18. L'Azou M, Moureau A, Sarti E, Nealon J, Zambrano B, Wartel TA, et al. Symptomatic Dengue in Children in 10 Asian and Latin American Countries. The New England journal of medicine. 2016;374(12):1155-66.
19. Thomas SJ, Nisalak A, Anderson KB, Libraty DH, Kalayanarooj S, Vaughn DW, et al. Dengue plaque reduction neutralization test (PRNT) in primary and secondary dengue virus infections: How alterations in assay conditions impact performance. Am J Trop Med Hyg. 2009;81(5):825-33.

20. Sirivichayakul C, Sabchareon A, Limkittikul K, Yoksan S. Plaque reduction neutralization antibody test does not accurately predict protection against dengue infection in Ratchaburi cohort, Thailand. Virol J. 2014;11:48.
21. WHO. The WHO Global Advisory Committee on Vaccine Safety GACVS Statement on Dengvaxia ® (CYDTDV), December 7, 2017 http://www.who.int/vaccine_safety/committee/GACVS-StatementonDengvaxia-CYD-TDV/en/ Accessed on 26 January 2018. 2017.
22. Department of Health. DOH puts dengue immunization on hold after new findings from Sanofi-Pasteur Manila: DOH; 2017 [cited 2017 5 December]; Available from: http://www.doh.gov.ph/node/11831.
23. WHO. WHO Expert Committee on Biological Standardization, sixty-second report. (WHO technical report series; no. 979) Annex 2. Geneva: World Health Organization; 2013 [cited 2017 22 January]. Available from: http://www.who.int/biologicals/expert_committee/TRS_979_62nd_report.pdf?ua=1.
24. de Alwis R, Smith SA, Olivarez NP, Messer WB, Huynh JP, Wahala WM, et al. Identification of human neutralizing antibodies that bind to complex epitopes on dengue virions. Proc Natl Acad Sci U S A. 2012;109(19):7439-44. Epub 2012/04/14.
25. Patel B, Longo P, Miley MJ, Montoya M, Harris E, de Silva AM. Dissecting the human serum antibody response to secondary dengue virus infections. PLoS Negl Trop Dis. 2017;11(5):e0005554. Epub 2017/05/16.
26. Vannice KS, Wilder-Smith A, Barrett ADT, Carrijo K, Cavaleri M, de Silva A, et al. Clinical development and regulatory points for consideration for second-generation live attenuated dengue vaccines. Vaccine. 2018;36(24):3411-7. Epub 2018/03/12.
27. Henein S, Swanstrom J, Byers AM, Moser JM, Shaik SF, Bonaparte M, et al. Dissecting Antibodies Induced by a Chimeric Yellow Fever-Dengue, Live-Attenuated, Tetravalent Dengue Vaccine (CYD-TDV) in Naive and Dengue-Exposed Individuals. J Infect Dis. 2017;215(3):351-8.
28. Weiskopf D, Angelo MA, de Azeredo EL, Sidney J, Greenbaum JA, Fernando AN, et al. Comprehensive analysis of dengue virus-specific responses supports an HLA-linked protective role for CD8+ T cells. Proc Natl Acad Sci U S A. 2013;110(22):E2046-53. Epub 2013/04/13.
29. Angelo MA, Grifoni A, O'Rourke PH, Sidney J, Paul S, Peters B, et al. Human CD4(+) T Cell Responses to an Attenuated Tetravalent Dengue Vaccine Parallel Those Induced by Natural Infection in Magnitude, HLA Restriction, and Antigen Specificity. J Virol. 2017;91(5). Epub 2016/12/16.
30. Nivarthi UK, Kose N, Sapparapu G, Widman D, Gallichotte E, Pfaff JM, et al. Mapping the Human Memory B Cell and Serum Neutralizing Antibody Responses to Dengue Virus Serotype 4 Infection and Vaccination. J Virol. 2017;91(5). Epub 2016/12/30.
31. de Silva AM, Harris E. Which Dengue Vaccine Approach Is the Most Promising, and Should We Be Concerned about Enhanced Disease after Vaccination? The Path to a Dengue Vaccine: Learning from Human Natural Dengue Infection Studies and Vaccine Trials. Cold Spring Harbor perspectives in biology. 2018;10(6). Epub 2017/07/19.
32. World Health Organization. Guidelines for plaque reduction neutralization testing of human antibodies to dengue viruses. 2007 [cited 2016 7 December]; Available from: http://apps.who.int/iris/handle/10665/69687.
33. Corbett KS, Katzelnick L, Tissera H, Amerasinghe A, de Silva AD, de Silva AM. Preexisting neutralizing antibody responses distinguish clinically inapparent and apparent dengue virus infections in a Sri Lankan pediatric cohort. J Infect Dis. 2015;211(4):590-9.
34. Kraus AA, Messer W, Haymore LB, de Silva AM. Comparison of plaque- and flow cytometry-based methods for measuring dengue virus neutralization. J Clin Microbiol. 2007;45(11):3777-80.
35. Notomi T, Mori Y, Tomita N, Kanda H. Loop-mediated isothermal amplification (LAMP): principle, features, and future prospects. J Microbiol. 2015;53(1):1-5.

36. Philippine National Health Research System. National Ethical Guidelines for Health Research. . Guidelines PHREBAHCftRotE, editor. Taguig City: Philippine Council for Health Research and Development; 2011.
37. David CC, Albert JRG. How has basic education in the Philippines fared and what else needs to be done? Policy Notes (Philippine Institute for Development Studies). 2015;8.

12 Annexes

Annex A. Data collection forms or other instruments used

Annex B. Informed consent and Assent forms

Annex C. Terms of reference

Annex C. Terms of Reference

This study will provide information on the effect of baseline dengue serostatus among tetravalent dengue vaccine CYD-TDV recipients on the risk of having subsequent virologically confirmed dengue. The Institute of Child Health and Human Development (ICHHD) at the University of the Philippines Manila – National Institutes of Health (UPM-NIH) in collaboration with the Department of Health (DOH) and the Research Institute for Tropical Medicine (RITM), will implement this study.

ICHHD

- Responsible for over-all implementation of the study
- Develop the study protocol and informed consent and assent forms
- Coordinate with DOH, RITM and hospital sites included in the study as well as other institutions/agencies that may be relevant in the conduct of the study (i.e. regional Department of Education, regional DOH offices, etc.)
- Coordinate all study-related activities in the field
- Ensure that the protocol complies with international and local ethical guidelines including approval from pertinent ethical review boards
- Prepare data collection forms, study SOPs and training materials
- Monitor study implementation in the field
- Hire and conduct training of staff
- Consolidate data collected from the study sites
- Analyze consolidated data
- Write up final report (including financial report) and manuscript of the study
- Coordinate with funding agency to ensure appropriate implementation and funding of the project
- Discuss and disseminate study results to different local and international scientific fora

DOH

- Provide support in coordinating with the Philippines regional, provincial and city health offices
- Provide technical support
- Assist in coordinating with local government units in the study implementation
- Assist in protocol development and report writing
- Assist ICHHD staff in ensuring proper conduct of the study
- Be the end user of the data as a guide in implementing school-based dengue vaccination

RITM

- Perform Dengue RT-PCR on serum samples of suspected dengue cases enrolled in the study
- Assist in protocol development and report writing
- Relay the results to the study team in a timely manner

University of North Carolina

- Will provide laboratory materials for the conduct of study
- Will perform neutralization and other immunologic tests of all sera, as appropriate
- Will perform cell-mediated and B-cell response analysis of immune response to dengue infection

GenBank numbers of the mature dengue virions

Serotype	Strain and Genotype	Genbank ID
**DENV1**	Sri Lanka 2012 Genotype I	AIE17470.1
**DENV2**	Sri Lanka 2016 Genotype Cosmopolitan	QBP33521.1
**DENV3**	Sri Lanka 1989 Genotype III	AFI55000
**DENV4**	Sri Lanka 1992 Genotype IIB	AHN50410

Supplementary Table 1. Characteristics of the participants by baseline dengue serostatus

Characteristics	Baseline dengue naïve (N=320)	Baseline dengue, monotypic profile (N=292)	Baseline dengue multitypic profile (N=2,384)	Total (N=2,996)	p-value*
	n (%)	n (%)	n (%)	n (%)	
Age (years) at enrollment					
Mean (SD)	10.6 (1.22)	10.6 (1.29)	11.0 (1.42)	10.9 (1.40)	<0.0001
Median (Min, Max)	10 (9, 14)	10 (9, 15)	11 (8, 15)	11 (8, 15)	
Sex					
Female	148 (46.25%)	153 (52.40%)	1244 (52.18%)	1545 (51.57%)	0.1311
Male	172 (53.75%)	139 (47.60%)	1140 (47.82%)	1451 (48.43%)	
Residence					
Bogo city	125 (39.06%)	129 (44.18%)	1304 (54.70%)	1558 (52.00%)	<0.0001
Balamban municipality	195 (60.94%)	163 (55.82%)	1080 (45.30%)	1438 (48.00%)	
Uses house screen‡					
Yes	25 (8.90%)	23 (8.98%)	248 (11.63%)	296 (11.09%)	0.2252
No	256 (91.10%)	233 (91.02%)	1884 (88.37%)	2373 (88.91%)	
Housing material‡					
Wood	127 (45.20%)	124 (48.44%)	1051 (49.30%)	1302 (48.78%)	0.4346
Cement/concrete	154 (54.80%)	132 (51.56%)	1081 (50.70%)	1367 (51.22%)	
Keeps containers and unused tires‡					
Yes	22 (7.83%)	30 (11.72%)	272 (12.76%)	324 (12.14%)	0.0501
No	259 (92.17%)	226 (88.28%)	1860 (87.24%)	2345 (87.86%)	
Number of individuals in the household‡					
Mean (SD)	5.6 (1.86)	5.8 (1.92)	5.7 (2.01)	5.7 (1.99)	0.6283
Median (Min, Max)	5 (2, 13)	5 (2, 13)	5 (1, 24)	5 (1, 24)	0.6601
Number of children within household‡					
Mean (SD)	2.7 (1.41)	2.8 (1.50)	2.6 (1.49)	2.7 (1.48)	0.2199
Median (Min, Max)	2 (0, 9)	2 (0, 9)	2 (0, 14)	2 (0, 14)	0.2583
Household head with > 6 years of schooling‡					
Yes	263 (93.59%)	247 (96.48%)	2032 (95.31%)	2542 (95.24%)	0.2954
No	18 (6.41%)	9 (3.52%)	100 (4.69%)	127 (4.76%)	
Migrated to current residence in the past 2 years‡					
Yes	4 (1.42%)	5 (1.95%)	61 (2.86%)	70 (2.62%)	0.3427
No	277 (98.58%)	251 (98.05%)	2071 (97.14%)	2599 (97.38%)	
Ownership of the following‡:					
Radio	107 (38.08%)	127 (49.61%)	932 (43.71%)	1166 (43.69%)	0.0267
Television	222 (79.00%)	187 (73.05%)	1578 (74.02%)	1987 (74.45%)	0.1688
Refrigerator	136 (48.40%)	143 (55.86%)	1081 (50.70%)	1360 (50.96%)	0.1949

Characteristics	Baseline dengue naïve (N=320)	Baseline dengue, monotypic profile (N=292)	Baseline dengue multitypic profile (N=2,384)	Total (N=2,996)	p-value*
	n (%)	n (%)	n (%)	n (%)	
Bicycle	91 (32.38%)	89 (34.77%)	596 (27.95%)	776 (29.07%)	0.0334
Motorcycle	155 (55.16%)	141 (55.08%)	1238 (58.07%)	1534 (57.47%)	0.4616
Mobile phone	276 (98.22%)	252 (98.44%)	2098 (98.41%)	2626 (98.39%)	0.9588
Desktop/handheld computer	43 (15.30%)	43 (16.80%)	346 (16.23%)	432 (16.19%)	0.8894
Car	15 (5.34%)	16 (6.25%)	115 (5.39%)	146 (5.47%)	0.8147
Ownership of all luxury items†‡:					
Yes	11 (3.91%)	12 (4.69%)	78 (3.66%)	101 (3.78%)	0.6466
No	270 (96.09%)	244 (95.31%)	2054 (96.34%)	2568 (96.22%)	
Ownership of at least 1 luxury item†‡:					
Yes	277 (98.58%)	255 (99.61%)	2113 (99.11%)	2645 (99.10%)	0.4665
No	4 (1.42%)	1 (0.39%)	19 (0.89%)	24 (0.90%)	
Estimated monthly household expenditure‡					
Mean (SD)	11298.9 (6402.54)	12101.6 (8200.76)	11573.2 (5995.39)	11595.0 (6282.84)	0.3146
Median (Min, Max)	10000 (2000, 60000)	10000 (2000, 100000)	10000 (1000, 80000)	10000 (1000, 100000)	0.2950
Use of topical insect repellant‡					
Yes	29 (10.32%)	18 (7.03%)	161 (7.55%)	208 (7.79%)	0.2448
No	251 (89.32%)	238 (92.97%)	1971 (92.45%)	2460 (92.17%)	0.1746
Don't know (Unknown)	1 (0.36%)	0 (0.00%)	0 (0.00%)	1 (0.04%)	0.2012
Use of burned mosquito coil‡					
Yes	31 (11.03%)	29 (11.33%)	297 (13.93%)	357 (13.38%)	0.2616
No	250 (88.97%)	227 (88.67%)	1835 (86.07%)	2312 (86.62%)	
Fogging in the past month‡					
Yes	1 (0.36%)	1 (0.39%)	5 (0.23%)	7 (0.26%)	0.4267
No	278 (98.93%)	254 (99.22%)	2126 (99.72%)	2658 (99.59%)	0.0653
Don't know (Unknown/NA)	2 (0.71%)	1 (0.39%)	1 (0.05%)	4 (0.15%)	0.0276

[Note] SD: Standard Deviation; Min: Minimum; Max: Maximum; N: total number of participants; n: number of participants who were observed in each category; N/A: Not Applicable.

* P-value has been derived using analysis of variance (ANOVA) and Kruskal-Wallis test for mean and median comparison, respectively for continuous variables, and chi-square test and Fisher's exact test for binary variables.

† Luxury items include a car, computer, mobile phone, refrigerator, TV.

‡ Missing information from N=327.

Supplementary Table 2. Dengue virus (DENV) serotypes during first episode of virologically-confirmed dengue

	Number of samples	%
DENV 1	28	18.3%
DENV 2	58	37.9%
DENV 3	52	34.0%
DENV 4	10	6.5%
DENV 1 and 3	1	0.7%
DENV 2 and 3	3	2.0%
DENV 3 and 4	1	0.7%
**Total RTPCR positive**	**153**	**100.0%**

Supplementary Table 3. Cumulative incidence rate (IR) of acute febrile illness and virologically confirmed dengue (VCD) in the cohort, by year of observation

Year(s)* of follow-up:	1		2		3		4		5		6	
	**Cases /N**	**IR/100PY (95% CI)**	**Cases /N**	**IR/100PY (95% CI)**	**Cases /N**	**IR/100PY (95% CI)**	**Cases /N**	**IR/100PY (95% CI)**	**Cases /N**	**IR/100PY (95% CI)I**	**Cases /N**	**IR/100PY (95% CI)**
**Acute febrile illness**	33 /2996	1.11 (0.79 ,1.55)	324 /2996	5.60 (5.04 ,6.23)	579 /2996	6.99 (6.46 ,7.56)	587 /2996	5.51 (5.09 ,5.96)	645 /2996	4.99 (4.63 ,5.38)	674 /2996	4.84 (4.50 ,5.21)
**VCD**	5 /2996	0.17 (0.07 ,0.40)	72 /2996	1.21 (0.96 ,1.53)	135 /2996	1.54 (1.30 ,1.82)	138 /2996	1.19 (1.01 ,1.40)	146 /2996	1.02 (0.87 ,1.20)	153 /2996	1.02 (0.87 ,1.19)
**VCD without warning signs**	2 /2996	0.07 (0.02 ,0.27)	34 /2996	0.57 (0.41 ,0.80)	62 /2996	0.71 (0.55 ,0.90)	63 /2996	0.54 (0.42 ,0.70)	68 /2996	0.47 (0.37 ,0.60)	73 /2996	0.48 (0.39 ,0.61)
**VCD with warning signs**	3 /2996	0.10 (0.03 ,0.31)	38 /2996	0.64 (0.47 ,0.88)	73 /2996	0.83 (0.66 ,1.04)	75 /2996	0.65 (0.52 ,0.81)	78 /2996	0.54 (0.44 ,0.68)	80 /2996	0.53 (0.43 ,0.66)
**Hospitalized VCD**	3 /2996	0.10 (0.03 ,0.31)	20 /2996	0.34 (0.22 ,0.52)	33 /2996	0.38 (0.27 ,0.53)	34 /2996	0.29 (0.21 ,0.41)	35 /2996	0.24 (0.18 ,0.34)	36 /2996	0.24 (0.17 ,0.33)

[Note] IR: Incidence Rate per 100 person-years (PY)
*Year(s) were calculated based on vaccination date for vaccinees and median vaccination date for non-vaccinees.

Supplementary Table 4. Incidence rate (IR) of acute febrile illness and virologically confirmed dengue (VCD) in the cohort, by dengue serostatus at baseline

Baseline dengue serostatus:	Naïve		Monotypic profile		Multitypic profile	
	**Cases /N**	**IR/100PY (95% CI)**	**Cases /N**	**IR/100PY (95% CI)**	**Cases /N**	**IR/100PY (95% CI)**
**Acute febrile illness**	81 /320	5.64 (4.56 ,6.96)	81 /292	6.15 (4.98 ,7.59)	512 /2384	4.59 (4.21 ,4.99)
**VCD**	28 /320	1.81 (1.25 ,2.61)	25 /292	1.74 (1.18 ,2.56)	100 /2384	0.83 (0.68 ,1.01)
**VCD without warning signs**	15 /320	0.97 (0.59 ,1.61)	9 /292	0.63 (0.33 ,1.20)	49 /2384	0.41 (0.31 ,0.54)
**VCD with warning signs**	13 /320	0.84 (0.49 ,1.44)	16 /292	1.11 (0.68 ,1.81)	51 /2384	0.42 (0.32 ,0.55)
**Hospitalized VCD**	4 /320	0.26 (0.10 ,0.69)	11 /292	0.76 (0.42 ,1.38)	21 /2384	0.17 (0.11 ,0.27)

[Note] IR: Incidence Rate per 100 person-years (PY)
